# Supplementary material for: Control of NAD+ homeostasis by autophagic flux modulates mitochondrial and cardiac function
Source: EMBO J. 2024 Jan 11;43(3):3. doi: 10.1038/s44318-023-00009-w (PMC10897141; doi:10.1038/s44318-023-00009-w)
Supplement: Supplementary file 1 — Appendix [file 44318_2023_9_MOESM1_ESM.pdf]

# APPENDIX

Control of $NAD^+$ homeostasis by autophagic flux modulates cardiac function

Quanjiang Zhang, Zhonggang Li, Qiuxia Li, Samuel A.J. Trammell, Mark S. Schmidt, Karla Maria Pires, Jinjin Cai, Yuan Zhang, Helena Kenny, Sihem Boudina, Charles Brenner, and E. Dale Abel

## TABLE OF CONTENTS

Appendix Figure S1. cAtg3-KO does not alter autophagic flux in non-cardiomyocyte tissues and does not influence MTOR and AMPK signaling in hearts . . . . . . . . . 2

Appendix Figure S2. cAtg3-KO does not alter protein expression of NADK, NAMPT or NAMNAT1-3 in hearts . . . . . . . . . 3

Appendix Figure S3. cAtg3-KO inhibited Sirt1 activities and increased protein acetylation in hearts . . . . . . . . . 4

Appendix Figure S4. cAtg3-KO does not alter PARP1/2 protein expression, PARP1/2 activities, or abundance of CD38 and BST1 (CD157) in hearts . . . . . . . . . 5

Appendix Figure S5. Oxygen consumption in response to increasing concentrations of Me-NAM in H9c2 cardiomyocytes . . . . . . . . . 6

Appendix Figure S6. Autophagosomes do not engulf NNMT protein or mRNA in H9c2 cardiomyocytes . . . . . . . . . 7

Appendix Table S1. Percentage of cAtg3 KO mice . . . . . . . . . 8

Appendix Table S2. Primer Sequences . . . . . . . . . 9-10

Appendix Table S3. Antibodies . . . . . . . . . 11

Appendix Table S4. siRNA sequence . . . . . . . . . 12

Appendix Table S5. Plasmids and adenovirus . . . . . . . . . 13

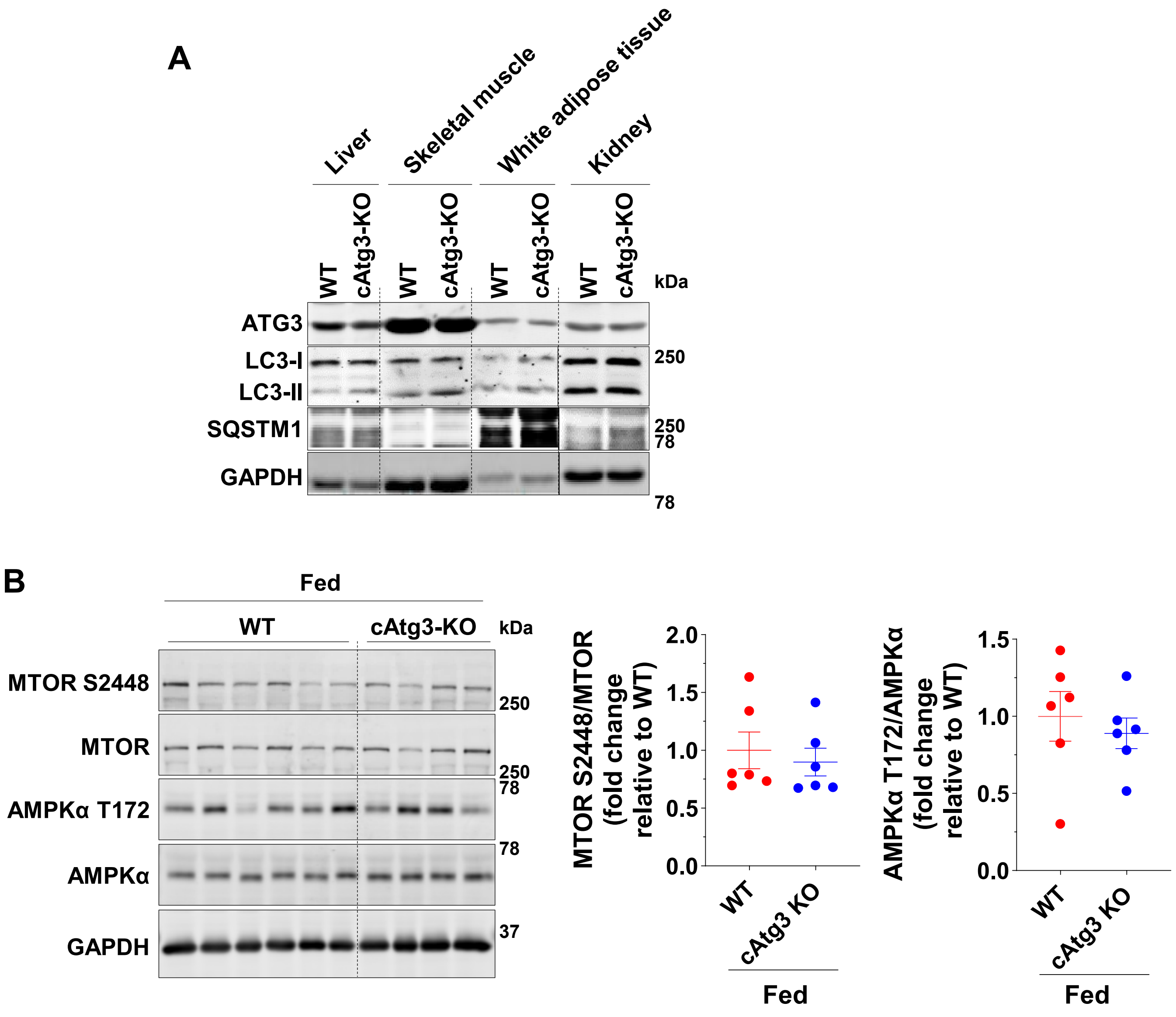


**Appendix Figure S1. cAtg3-KO does not alter autophagic flux in non-cardiomyocyte tissues and does not influence MTOR and AMPK signaling in hearts, Related to Figure 1.**

A Protein levels of ATG3, LC3 (MAP1LC3A), SQSTM1, and GAPDH in liver, skeletal muscle, white adipose tissue, and kidney of WT and cAtg3-KO mice. Representative blots are shown, n=2 per group.

B Quantification of MTOR S2448 and AMPKα T172 phosphorylation. Mice were randomly fed. n=6 per group. Data are mean ± SEM.

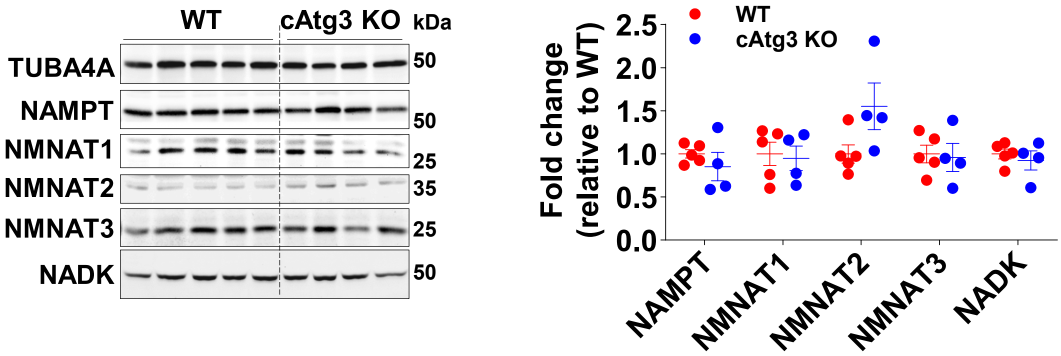


**Appendix Figure S2. cAtg3-KO does not alter protein expression of NADK, NAMPT or NAMNAT1-3 in hearts, Related to Figure 5.**

Protein levels of NADK, NAMPT, and NAMNAT1-3, TUBA4A (alpha-tubulin) in WT and cAtg3 KO mouse hearts. Mice were randomly fed, n=4 to 5 per group. Data are mean ± SEM. Representative section images are shown.

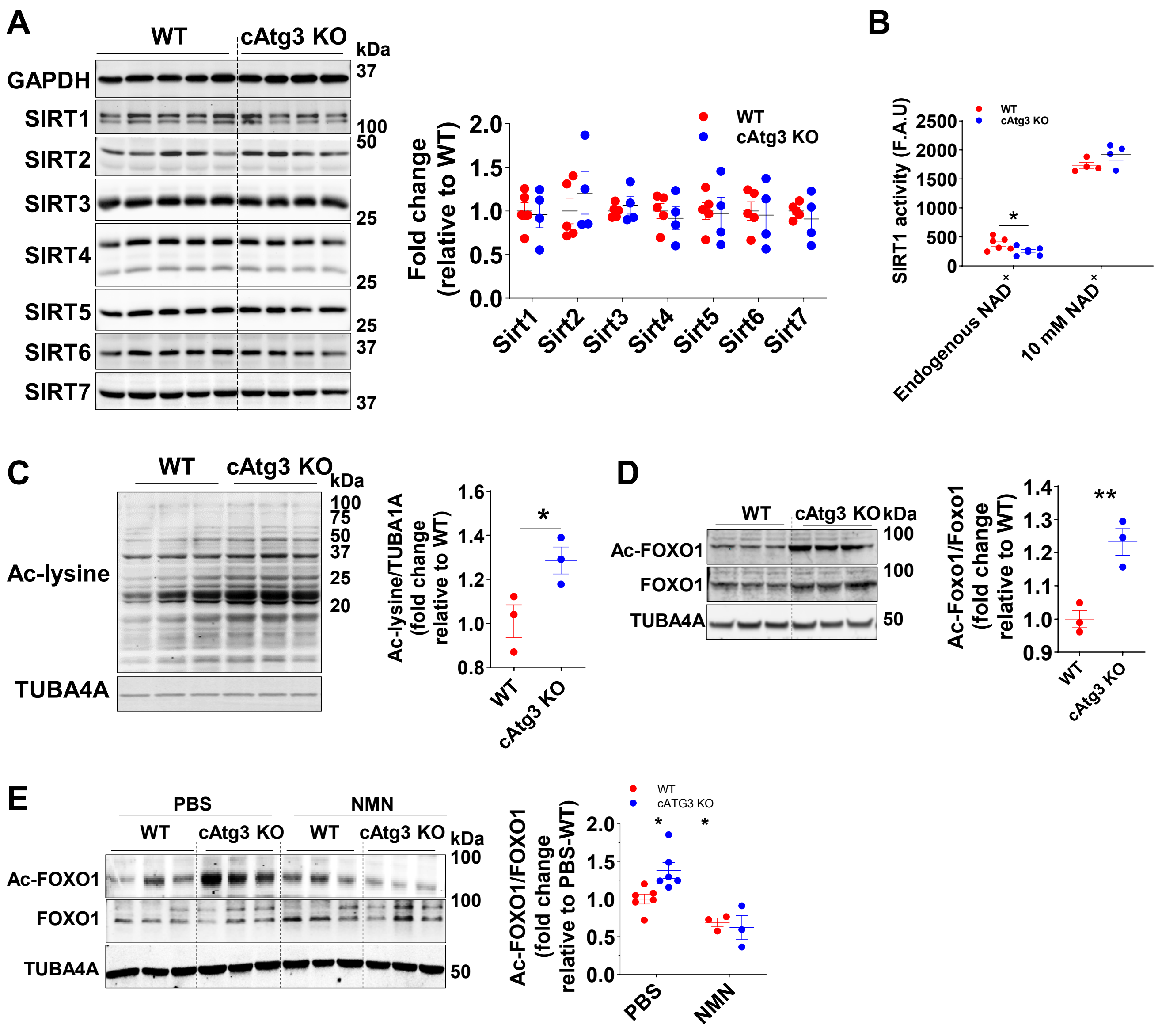


**Appendix Figure S3. cAtg3-KO inhibited Sirt1 activities and increased protein acetylation in hearts, Related to Figure 5.**

A Protein levels of SIRT1 to 7 and GAPDH in WT and cAtg3 KO mouse hearts. Mice were randomly fed, n=4 to 5 per group. Data are mean ± SEM.

B SIRT1 enzymatic activity measurement in 4-week-old WT and Atg3 KO mouse hearts, without or with the addition of 10 mM exogenous $NAD^+$, n=4 to 6 per group. Data are mean ± SEM. Unpaired t-tests were used to determine statistical significance between two groups. *p<0.05.

C Levels of Ac-lysine and TUBA4A (alpha-tubulin) in 4-week-old WT and cAtg3 KO mouse hearts. Mice were randomly fed, n=3 per group. Data are mean ± SEM. An unpaired t-test was used to determine statistical significance between two groups. *p<0.05.

D Protein levels of Ac-FOXO1, FOXO1, and TUBA4A in 4-week-old WT and cAtg3 KO mouse hearts. Mice were randomly fed, n=3 per group. Data are mean ± SEM. An unpaired t-test was used to determine statistical significance between two groups. **p<0.01.

E Ac-FOXO1 expression levels in hearts from mice that were injected with PBS or NMN, n=3 to 6 per group. Data are mean ± SEM. One-way ANOVA followed by Bonferroni's multiple comparison tests was used to determine statistical significance. *p<0.05.

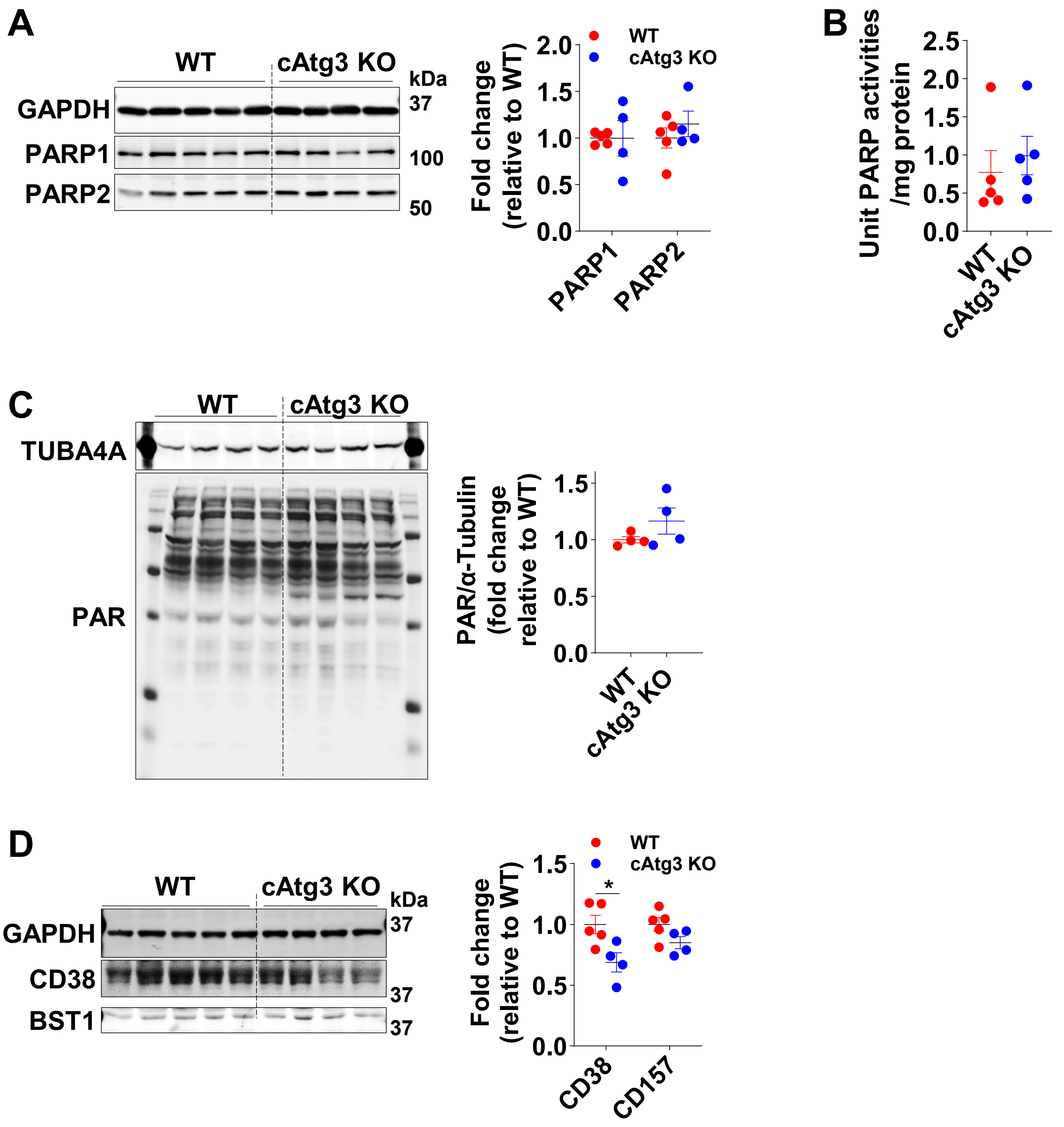


**Appendix Figure S4. cAtg3-KO does not alter PARP1/2 protein expression, PARP1/2 activities, or abundance of CD38 and BST1 (CD157) in hearts, Related to Figure 5.**

A PARP1/2 protein expression in 4-week-old WT and cAtg3 KO mouse hearts, n=4 to 5 per group. Data are mean ± SEM.

B PARP1/2 enzymatic activity measurement in 4-week-old WT and cAtg3 KO mouse hearts, n=5 per group. Data are mean ± SEM.

C Abundance of PAR in 16-week-old WT and cAtg3 KO mouse hearts, n=4 per group. Data are mean ± SEM.

D Protein levels of CD38 and BST1, and GAPDH in WT and cAtg3 KO mouse hearts. Mice were randomly fed, n-4 to 5 per group. Data are mean ± SEM. An unpaired t-test was used to determine statistical significance between two groups. *p<0.05. Representative section images are shown.

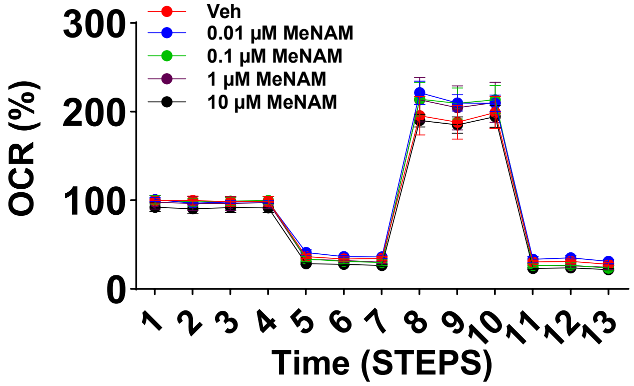


**Appendix Figure S5. Oxygen consumption in response to increasing concentrations of Me-NAM in H9c2 cardiomyocytes, Related to Figure 6.**
Oxygen consumption rate (OCR) in H9c2 cells treated with PBS (Veh) or concentrations of MeNAM as indicated, n=3 to 4 per group. Data are mean ± SEM. Step 1 to 4: 20 μM glucose medium, step 5 to 7: oligomycin (1μg/ml), step 8 to 10: FCCP (0.5μg/ml), step 11 to 13: rotenone (1μg/ml).

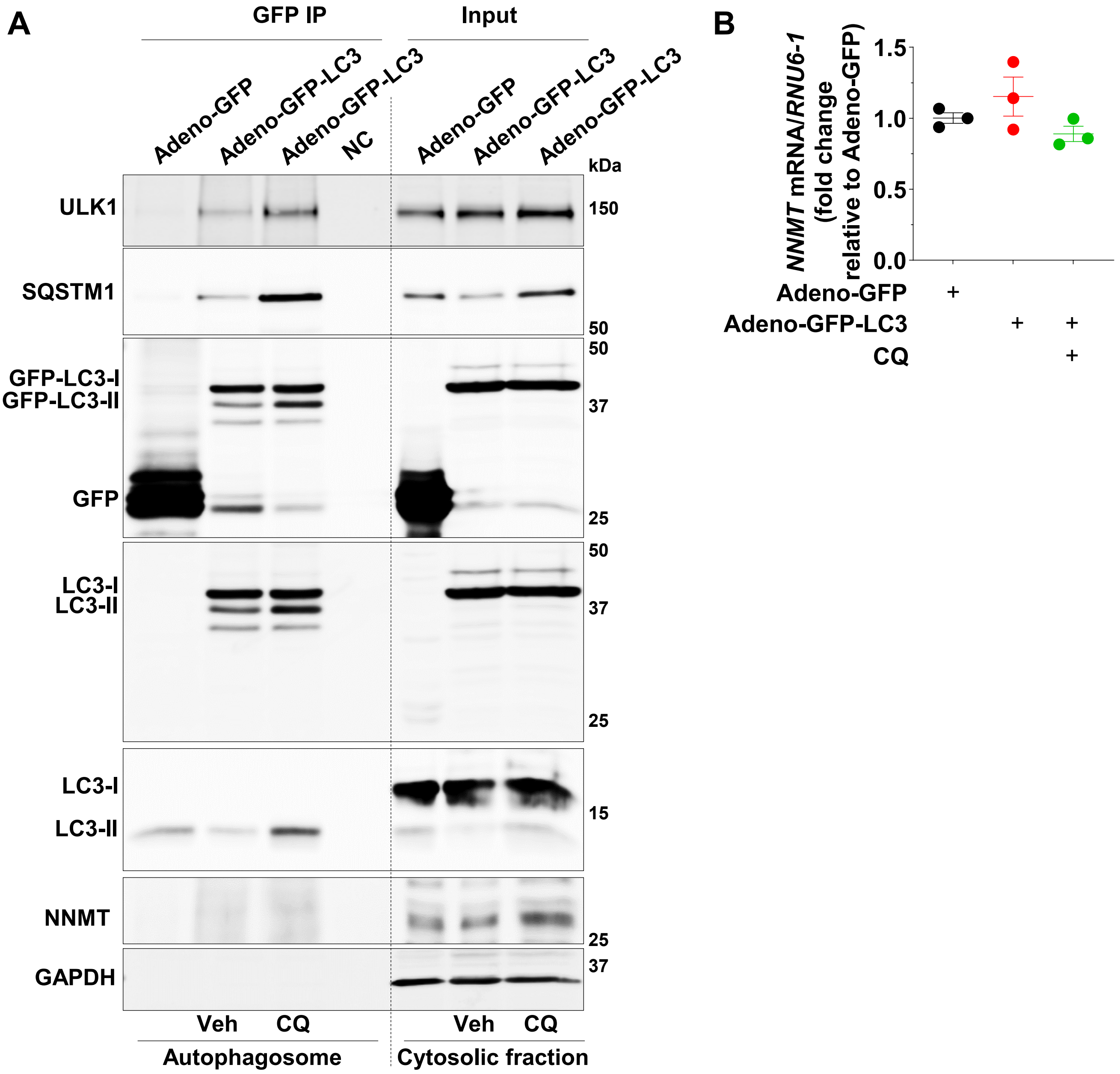


**Appendix Figure S6. Autophagosomes do not engulf NNMT protein or mRNA in H9c2 cardiomyocytes, Related to Figure 7.** H9c2 cardiomyocytes were first transfected with adenovirus encoding either GFP or GFP-tagged LC3 (MAP1LC3A), and then cells encoding GFP-LC3 were treated with either vehicle (Veh) or chloroquine (CQ) at 20 uM for 16 h. under nutrient replete conditions.

A The protein levels of ULK1, SQSTM1, GFP, GFP-LC3, LC3, NNMT, and GAPDH in isolated autophagosomes. GFP-LC3 were probed with antibodies against GFP and LC3, and the images of immunoblots were acquired using LI-COR Odyssey DXL system at two independent laser channels, respectively. Therefore, GFP antibody detects GFP and GFP-LC3, and LC3 antibody detects GFP-LC3 only. The experiments were repeated three times. Representative blots are shown. NC indicates the separation buffer with GFP antibody. Input is cytosolic fractions.

B *NNMT* mRNA levels in isolated autophagosomes. n=3 per group. Data are mean±SEM.

## Appendix Tables

Table S1. **Percentage of cAtg3 KO mice**

| Sex | Genotype | N. | % |
|---|---|---|---|
| M | WT | 89 | 25.72 |
| | cAtg3 KO | 85 | 24.57 |
| F | WT | 81 | 23.41 |
| | cAtg3 KO | 91 | 26.30 |

**Appendix Tables**

Table S2. **Primer Sequences**

| Gene | Sequences |
|---|---|
| *GAPDH* (M) | GCAACAATCTCCACTTTGCCAC<br>AATGGTGAAGGTCGGTGTGAAC |
| *GAPDH* (R) | GCTCTCTGCTCCTCCCTGTTC<br>GAGGCTGGCACTGCACAA |
| *RP16S* | GATTTGCTGGTGTGGATATC<br>TCTTTGATCTCCTTCTTAGA |
| *ACTB* (M) | CGATGCCCTGAGGCTCTT T<br>TGGATGCCACAGGATTCCA |
| *ACTB* (R) | GAGACCTTCAACACCCCAGCC<br>TCGGGGCATCGGAACCGCTCA |
| *RPL13A* (M) | CTCTGGCCTTTTCCTTTTTG<br>CCGAAGAAGGGAGACAGTTC |
| *RPL13A* (R | CCACCCTATGACAAGAAAAAGC<br>ACATTCTTTTCTGCCTGTTTCC |
| *18S* (R) | GCCGCTAGAGGTGAAATTCTTA<br>CTTTCGCTCTGGTCCGTCTT |
| *RNU6-1* (R) | GCAAATTCGTGAAGCGTTCC |
| *NPPA* (M) | ATGGGCTCCTTCTCCATCA<br>CCTGCTTCCTCAGTCTGCTC |
| *NPPB* (M) | GGATCTCCTGAAGGTGCTGT<br>TTCTTTTGTGAGGCCTTGGT |
| *MYH7* (M) | GCCATCATGCACTTTGGAAAC<br>CCCATGAGGTAGGCTGATTTGT |
| *PLN* (M) | AACAGGCAGCCAAATGTGA<br>CCCAGCTAAGCTCCCATAAG |
| *NNMT* (R) | CAGAGCTGAGACACGATGGA<br>GCAGGCAGAGAGAAGCTGAT |
| *NNMT* (M) | GATTGCACGCCTCAACTTCT<br>GAACCAGGAGCCTTTGACTG |
| *NRF1* (M) | CTTCAGAACTGCCAACCACA<br>GCTTCTGCCAGTGATGCTAC |
| *NRF2* (M) | AGTCTTCACTGCCCCTCATC<br>TCTGTCAGTGTGGCTTCTGG |
| *TFAM* (M) | GCAAAGGATGATTCGGCTC<br>TCTGCTCTTCCCAAGACTTCA |
| *NMNAT1* (M) | TGAGTCCATGGGGAGAAGTT<br>AGGACTAGGGCCGTTTGG |
| *NMNAT2* (M) | ATCCCGCCAATCACAATAAA<br>GCAGCTTCAATCCCATCACT |
| *NMNAT3* (M) | CAGAAGCACCACAGGGATTC<br>CCTGCAGCACGTTTACAGTC |
| *NMAPT* (M) | TCACGGCATTCAAAGTAGGA<br>GCAGAAGCCGAGTTCAACAT |
| *NMRK1* (M) | CTTGAAGCTTGCTCTGCGAC<br>CTCCGTTTGTCACACCACCA |
| *NMRK2* (M) | AAGCCCCAGGACCAAATAGC<br>GCGTGCAAACTTGTGTGGAT |

| | |
|---|---|
| *PARP1* (M) | CACCTTCCAGAAGCAGGAGA<br>GCAGCGAGAGTATTCCCAAG |
| *PARP2* (M) | GCAACAGAAGACGACTCTCCT<br>CAGCCATAGGCCCTTTTCTCT |
| *RPL32* | ACATCGGTTATGGGAGCAAC<br>GGGATTGGTGACTCTGATGG |
| *mtDNA* (M) | CCTATCACCCTTGCCATCAT<br>GAGGCTGTTGCTTGTGTGAC |
| *CHROMOSOME 6* (M) | ATGGAAAGCCTGCCATCATG<br>TCCTTGTTGTTCAGCATCAC |
| *PPARa* | GAGAATCCACGAAGCCTACC<br>AATCGGACCTCTGCCTCTTT |
| *VLCAD* | AGGCAGTTCTGGACAAGCCA<br>TTCCTCAAAGAACCGGGCCA |
| *LCAD* | GCATTGGTGGGGACTTGCTC<br>TGTCATGGCTATGGCACCGA |
| *MCAD* | ACTGACGCCGTGCAGATTTT<br>GCTTAGTTACACGAGGGTGATG |

**Appendix Tables**

Table S3. **Antibodies**

| Name | Company | Catalog Number |
|---|---|---|
| ATG3 | Sigma-Aldrich | A3231 |
| LC3 | Sigma-Aldrich | L8918 |
| TUBA4A | Sigma-Aldrich | T5168 |
| ACTB | Cell Signaling | 3700 |
| SQSTM1 | Cell Signaling | 5114 |
| Acetylated-Lysine | Cell Signaling | 9441 |
| ATG7 | Cell Signaling | 8858 |
| FOXO1 | Cell Signaling | 2880 |
| RELA S536 | Cell Signaling | 3036 |
| RELA | Cell Signaling | 6956 |
| MTOR S2448 | Cell Signaling | 4517 |
| MTOR | Cell Signaling | 2971 |
| P70S6K T389 | Cell Signaling | 9206 |
| p70S6K | Cell Signaling | 2708 |
| AMPKα T172 | Cell Signaling | 2531 |
| AMPKα | Cell Signaling | 2793 |
| ULK1 | Cell Signaling | 8054 |
| GFP | Cell Signaling | 55494 |
| Sirtuin antibody sampler kit | Cell Signaling | 9787 |
| SIRT4 | Cell Signaling | 69786 |
| PARP1 | Cell Signaling | 9532 |
| NADK | Cell Signaling | 89833 |
| NRF1 | Cell Signaling | 69432 |
| GAPDH | Cell Signaling | 2118 |
| PRKN | Cell Signaling | 4211 |
| CASP3 | Cell Signaling | 9665 |
| CD38 | Proteintech | 60006-1-Ig |
| BST1 | Proteintech | 16337-1-AP |
| NMNAT1 | Proteintech | 11399-1-AP |
| NMNAT3 | Proteintech | 13236-1-AP |
| PARP2 | Proteintech | 20555-1-AP |
| NMNAT2 | ThermoFisher Scientific | PA5-115662 |
| NNMT | Proteintech | 15123-1-AP |
| PINK1 | Proteintech | 23274-1-AP |
| NAMPT | Proteintech | 11776-1-AP |
| GAPDH | SCBT | SC-32233 |
| TFAM | SCBT | SC-166965 |
| NRF2 | SCBT | SC-722 |
| Ac-FKHR | SCBT | SC-49437 |
| PARGC1A | SCBT | SC-13067 |
| SIRT1 | SCBT | SC-74504 |
| TOMM20 | SCBT | SC-17764 |
| SDHA | abcam | ab14715 |

## Appendix Tables

Table S4. **siRNA sequence**

| Name | Sequences/Company with catalog number |
|---|---|
| Control siRNA | 5´-UAAGGCUAUGAAGAGAUAC-3’<br>5’-GUAUCUCUUCAUAGCCUUA-3’ |
| *ATG3* siRNA | 5’-CCCAGAAGAGUUUGUGGCAGCUGGA-3’<br>5’-UCCAGCUGCCACAAACUCUUCUGGG-3’ |
| ON-TARGETplus SMARTpool *NNMT* siRNA | GE Dharmacon (L-101014-02-0005) |
| SignalSilence *SQSTM1/p62* siRNA II | Cell Signaling Technology (6399) |
| *SQSTM1/p62* | 5’-CCUGUGGUGGGAACUCGCUAUAAGU-3’<br>5’-ACUUAUAGCGAGUUCCCACCACAGG-3’ |
| SignalSilence® *NF-κB p65* (*RELA*) siRNA I | Cell Signaling Technology (6261S) |

## Appendix Tables

Table S5. **Plasmids and adenovirus**

| | Name | Company/Institute | Catalog Number/Provider |
|---|---|---|---|
| Plasmids | *HA-FLAG* | Addgene | 10792 |
| | *HA-SQSTM1* | Addgene | 28027 |
| | *ATG7* | Addgene | 24921 |
| Adenovirus | *GFP* | Beth Israel Deaconess Medical Center, Boston, MA | Dr. Pavlos Pissios |
| | *GFP-LC3* | Cedars Sinai, Los Angeles, CA | Dr. Roberta A. Gottlieb |
| | *NNMT* | Beth Israel Deaconess Medical Center, Boston, MA | Dr. Pavlos Pissios |
