# Supplementary figures and images for: Control of NAD+ homeostasis by autophagic flux modulates mitochondrial and cardiac function
Source: EMBO J. 2024 Jan 11;43(3):3. doi: 10.1038/s44318-023-00009-w (PMC10897141; doi:10.1038/s44318-023-00009-w)

Fig. 1

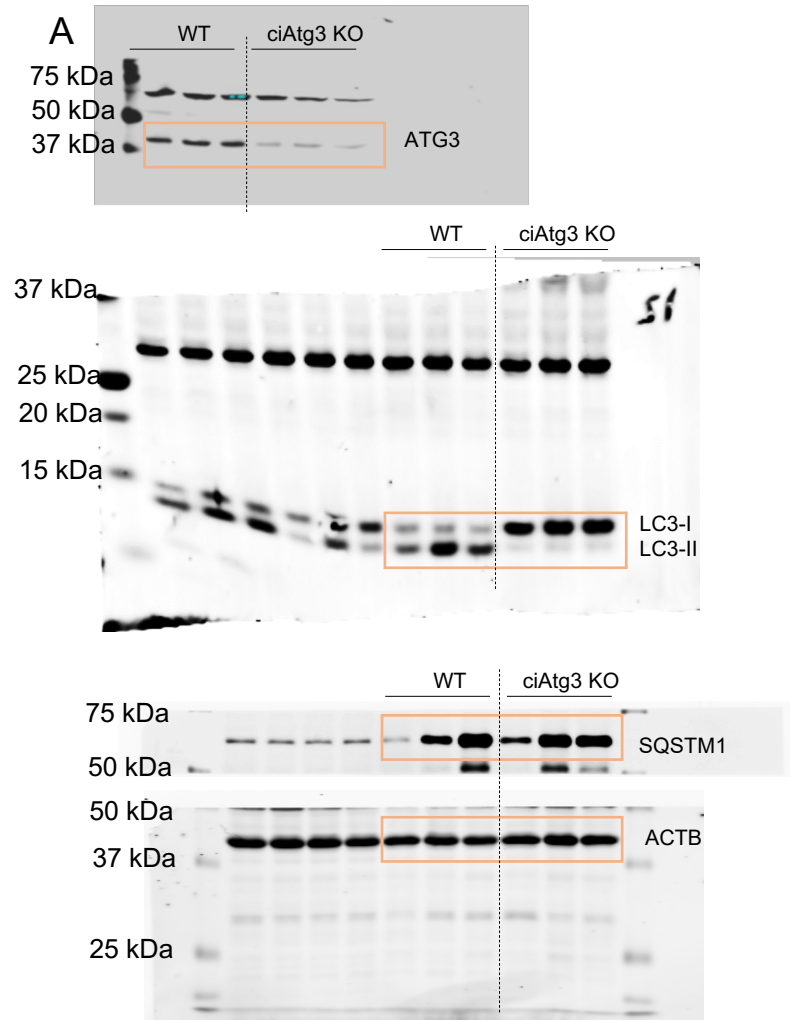

Supplement: Supplementary file 2 — Source Data Fig. 1 [file 44318_2023_9_MOESM2_ESM.zip › Figure 1/1 A/1 A Image data-blot.pdf]

1 F

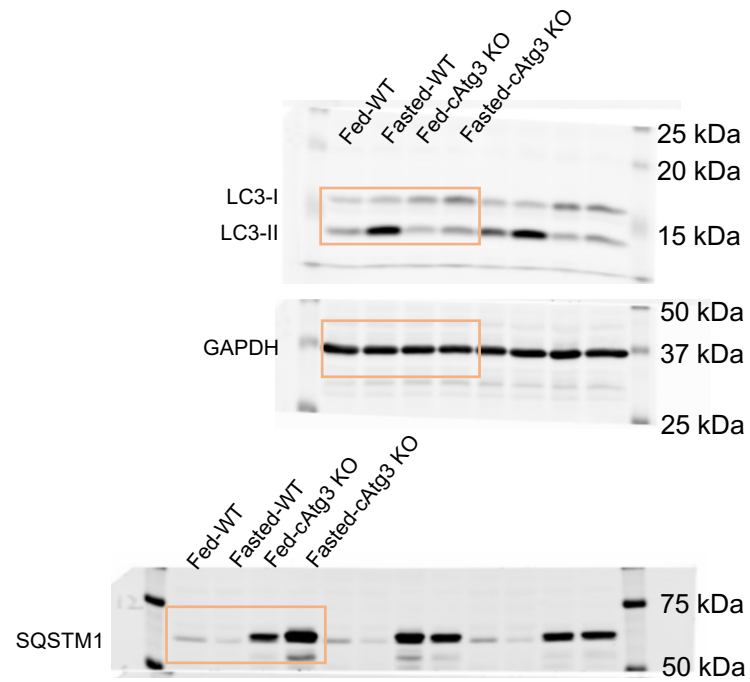

Supplement: Supplementary file 2 — Source Data Fig. 1 [file 44318_2023_9_MOESM2_ESM.zip › Figure 1/1 F/1F Image blot.pdf]

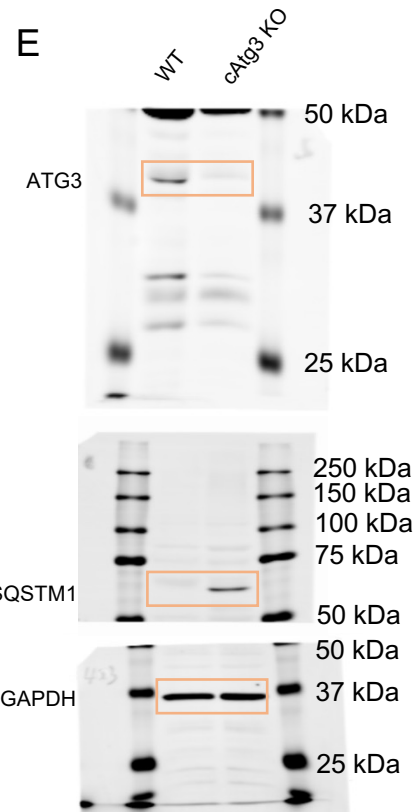

Supplement: Supplementary file 2 — Source Data Fig. 1 [file 44318_2023_9_MOESM2_ESM.zip › Figure 1/1 E/1 E Image data-Blot.pdf]

Fig. 2

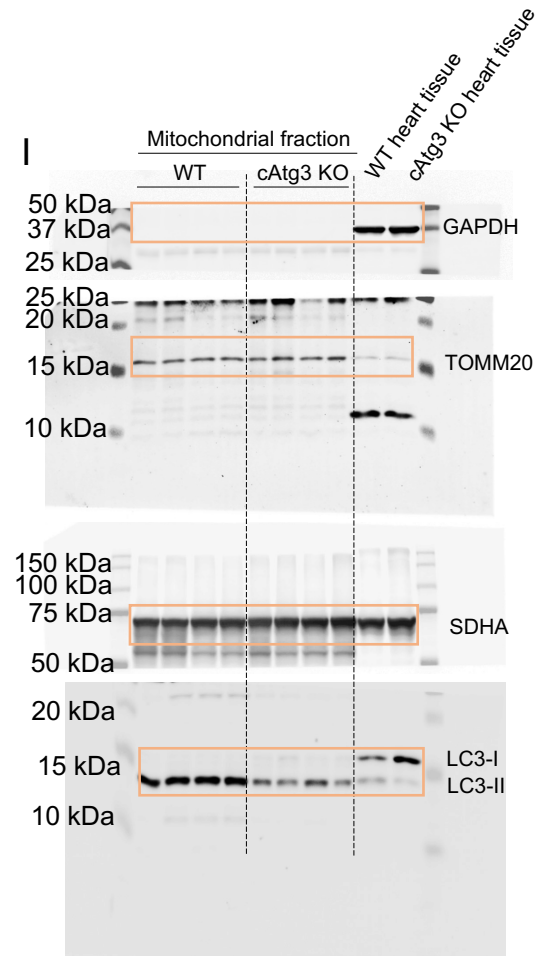

Supplement: Supplementary file 3 — Source Data Fig. 2 [file 44318_2023_9_MOESM3_ESM.zip › Figure 2/2I/2I Image data-Blot .pdf]

Fig. 2

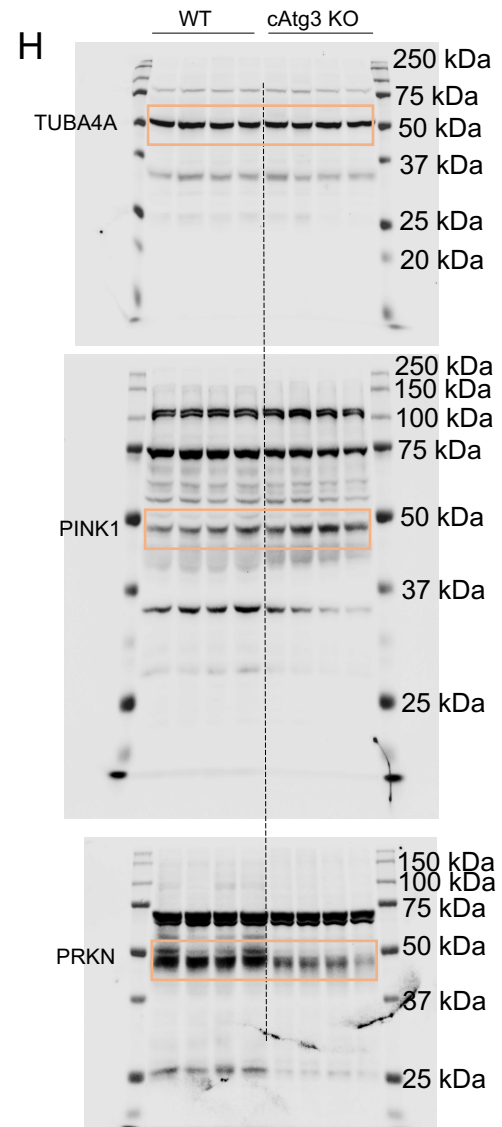

Supplement: Supplementary file 3 — Source Data Fig. 2 [file 44318_2023_9_MOESM3_ESM.zip › Figure 2/2H/2H Image data- Blot.pdf]

Fig. 2

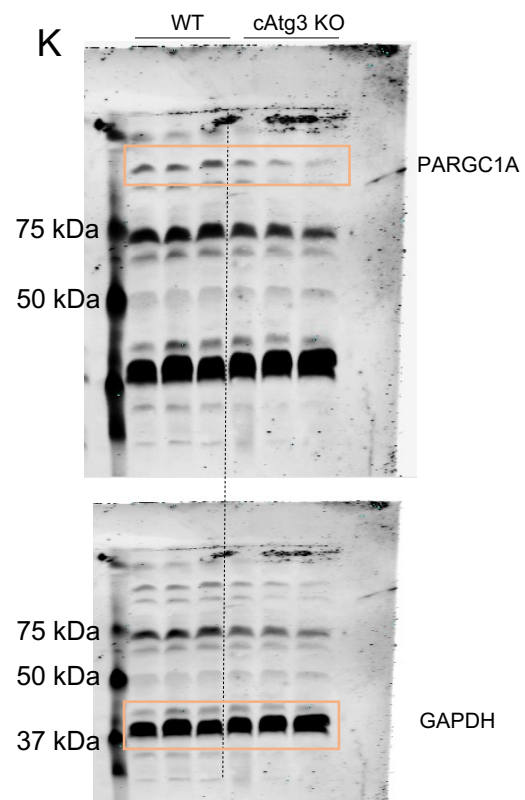

Supplement: Supplementary file 3 — Source Data Fig. 2 [file 44318_2023_9_MOESM3_ESM.zip › Figure 2/2K/2K Image data -Blot.pdf]

Fig. 2

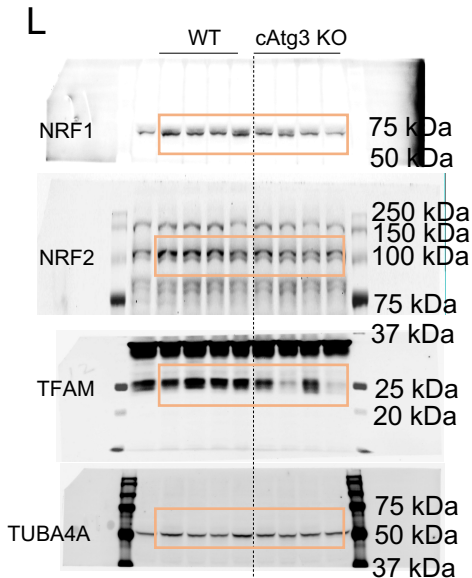

Supplement: Supplementary file 3 — Source Data Fig. 2 [file 44318_2023_9_MOESM3_ESM.zip › Figure 2/2L/2L Image data- Blot.pdf]

Fig. 3 C

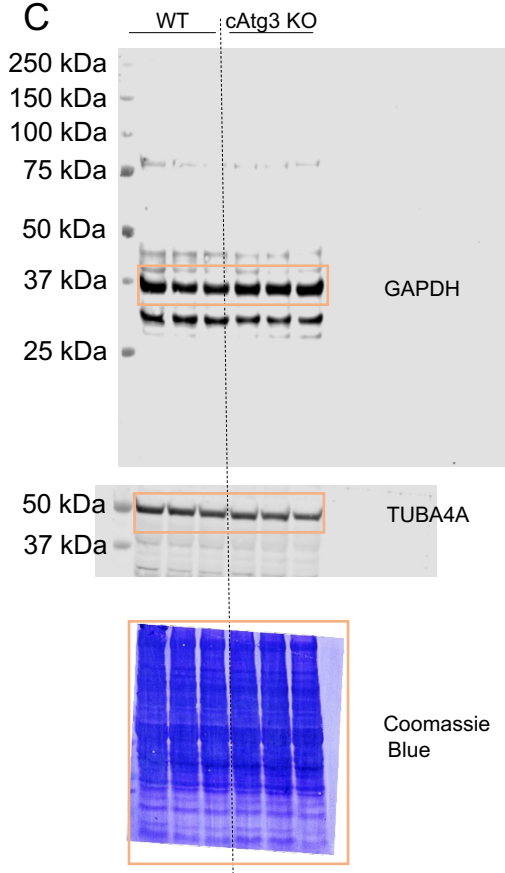

Supplement: Supplementary file 4 — Source Data Fig. 3 [file 44318_2023_9_MOESM4_ESM.zip › Figure 3/3C/3C Image data-blot.pdf]

Fig 4

A

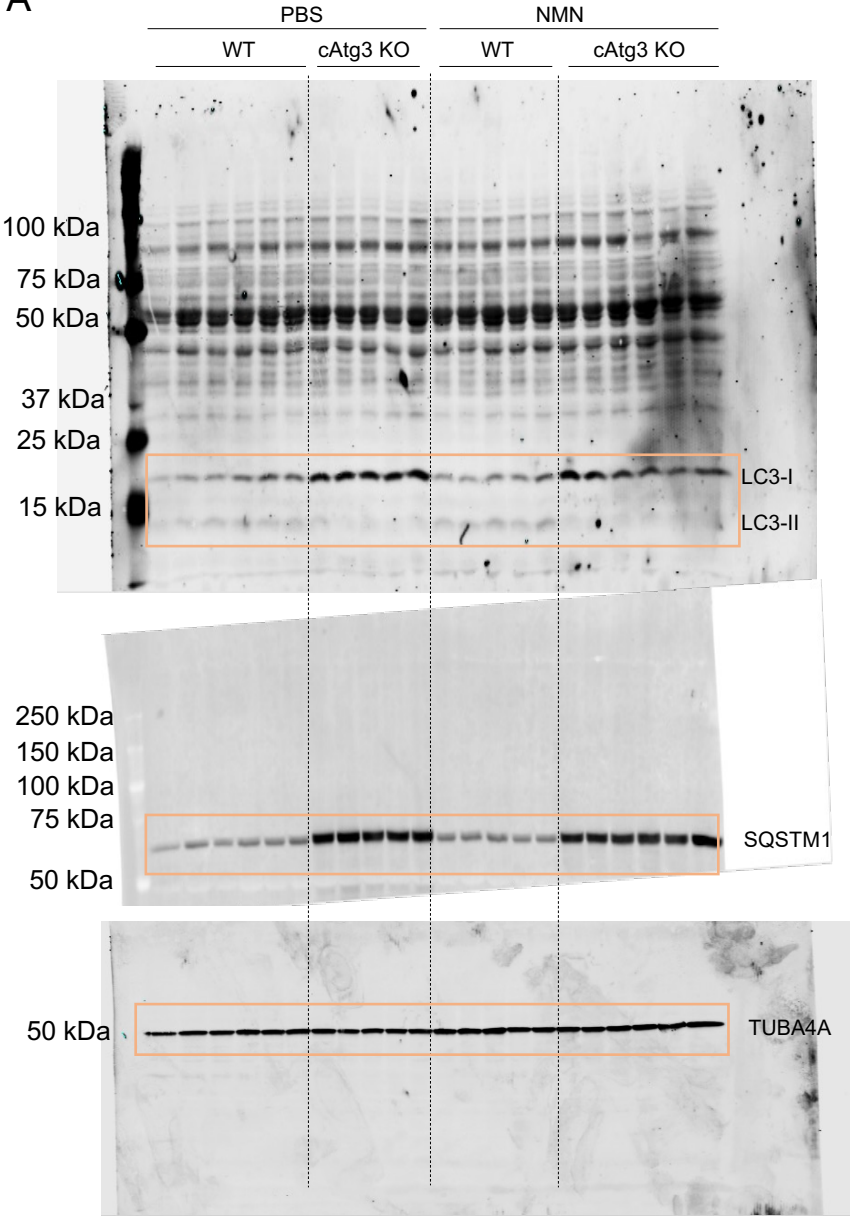

Supplement: Supplementary file 5 — Source Data Fig. 4 [file 44318_2023_9_MOESM5_ESM.zip › Figure 4/4A/4A Image data-Blot.pdf]

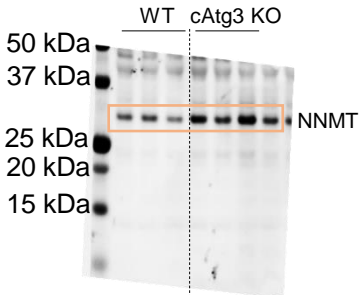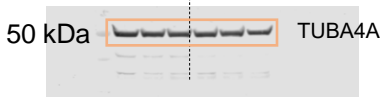

Supplement: Supplementary file 6 — Source Data Fig. 5 [file 44318_2023_9_MOESM6_ESM.zip › Figure 5/5G/Image-Blot.pdf]

Ad-GFP      Ad-NNMT

37 kDa

25 kDa

NNMT

150 kDa

100 kDa

75 kDa

50 kDa

TUBA4A

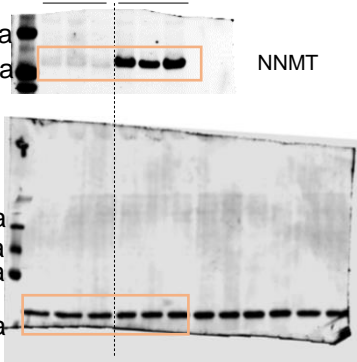

Supplement: Supplementary file 6 — Source Data Fig. 5 [file 44318_2023_9_MOESM6_ESM.zip › Figure 5/5H/Image-Blot.pdf]

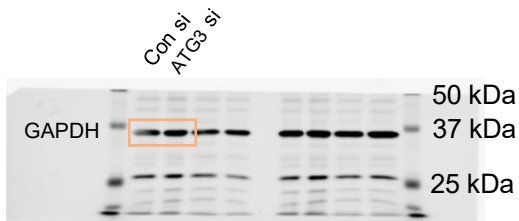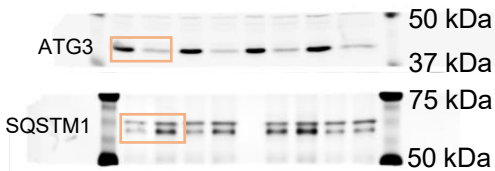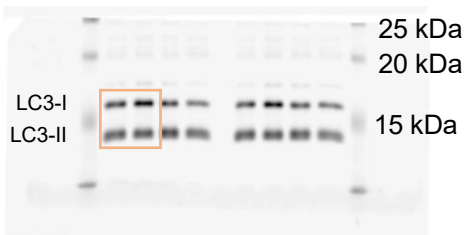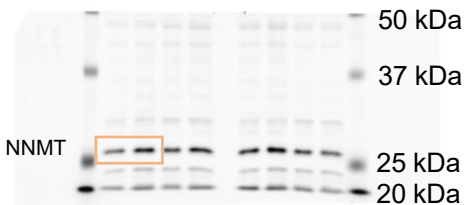

Supplement: Supplementary file 7 — Source Data Fig. 6 [file 44318_2023_9_MOESM7_ESM.zip › Figure 6/6A/Image-Blot.pdf]

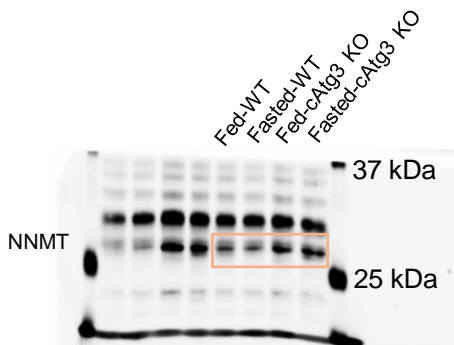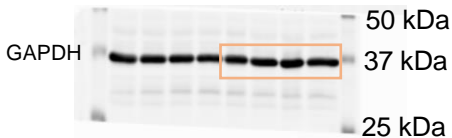

Supplement: Supplementary file 7 — Source Data Fig. 6 [file 44318_2023_9_MOESM7_ESM.zip › Figure 6/6I/Image-Blot.pdf]

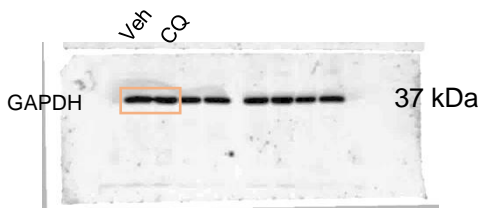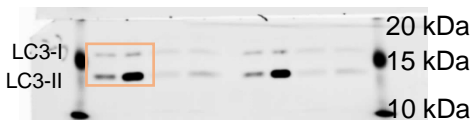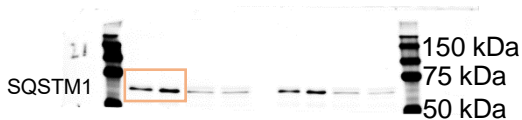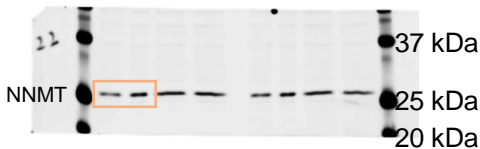

Supplement: Supplementary file 7 — Source Data Fig. 6 [file 44318_2023_9_MOESM7_ESM.zip › Figure 6/6E/Image-Blot.pdf]

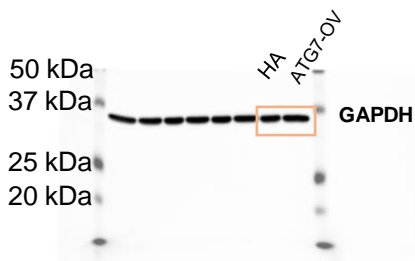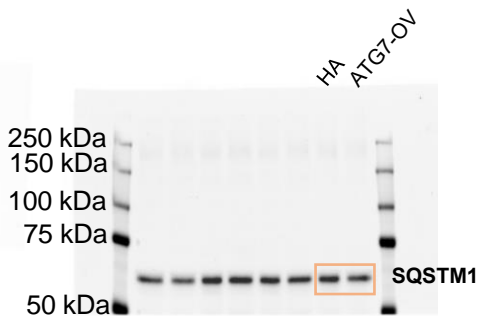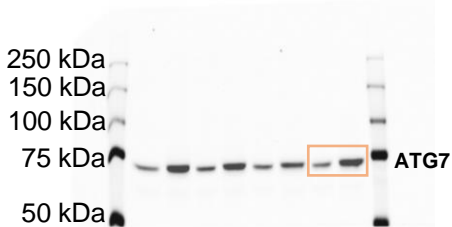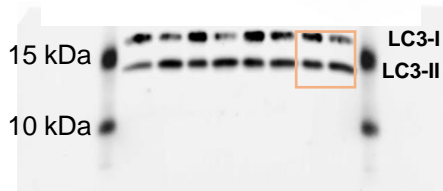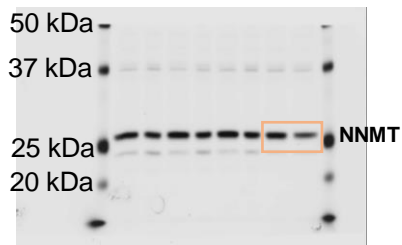

Supplement: Supplementary file 7 — Source Data Fig. 6 [file 44318_2023_9_MOESM7_ESM.zip › Figure 6/6K/Image-Blot.pdf]

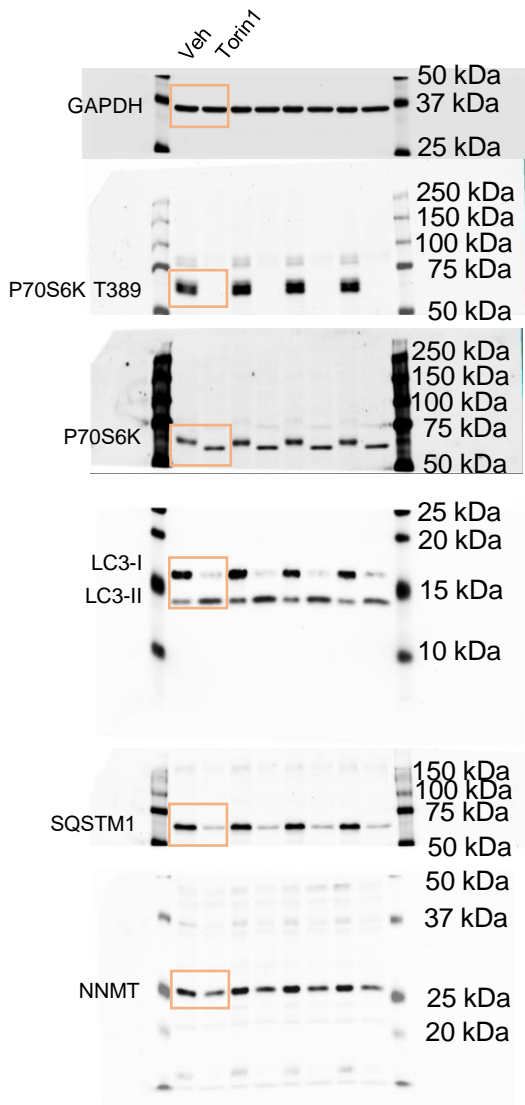

Supplement: Supplementary file 7 — Source Data Fig. 6 [file 44318_2023_9_MOESM7_ESM.zip › Figure 6/6M/Image-Blot.pdf]

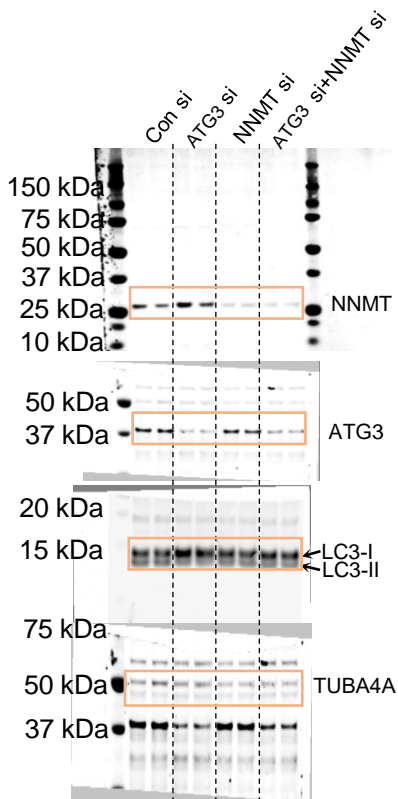

Supplement: Supplementary file 7 — Source Data Fig. 6 [file 44318_2023_9_MOESM7_ESM.zip › Figure 6/6C/Image-Blot.pdf]

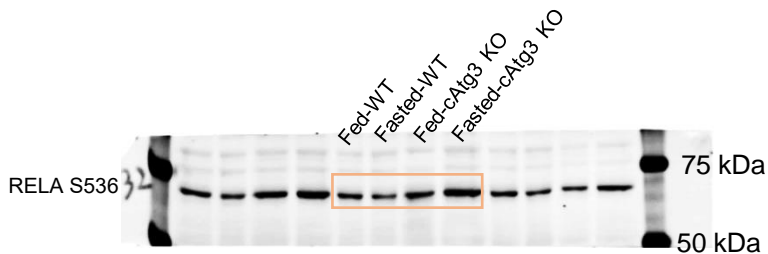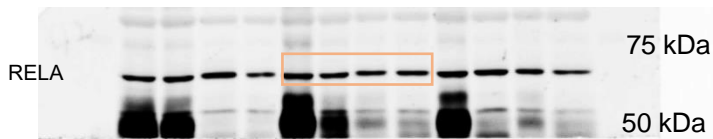

Supplement: Supplementary file 8 — Source Data Fig. 7 [file 44318_2023_9_MOESM8_ESM.zip › Figure 7/7D/Image-Blot.pdf]

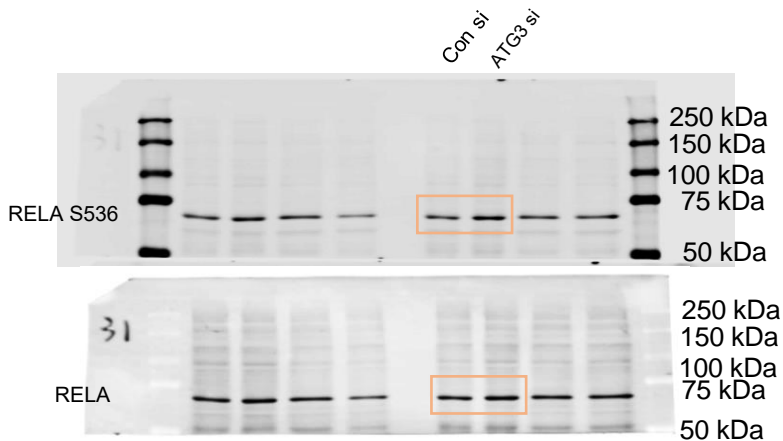

Supplement: Supplementary file 8 — Source Data Fig. 7 [file 44318_2023_9_MOESM8_ESM.zip › Figure 7/7C/Image-Blot.pdf]

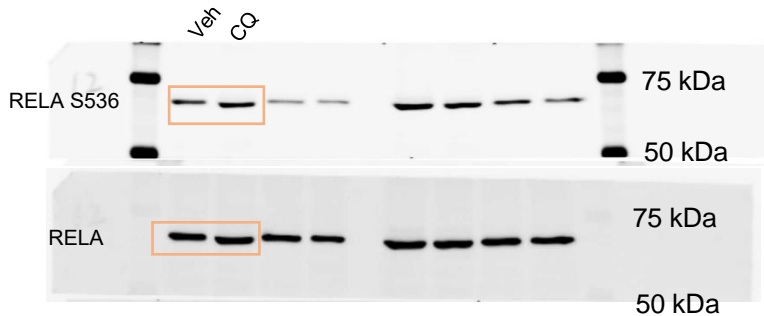

Supplement: Supplementary file 8 — Source Data Fig. 7 [file 44318_2023_9_MOESM8_ESM.zip › Figure 7/7B/Image-Blot.pdf]

1000  
500  
400  
300  
200  
100

NNMT Promoter

Input  
RELA  
Histone H3  
IgG  
No DNA

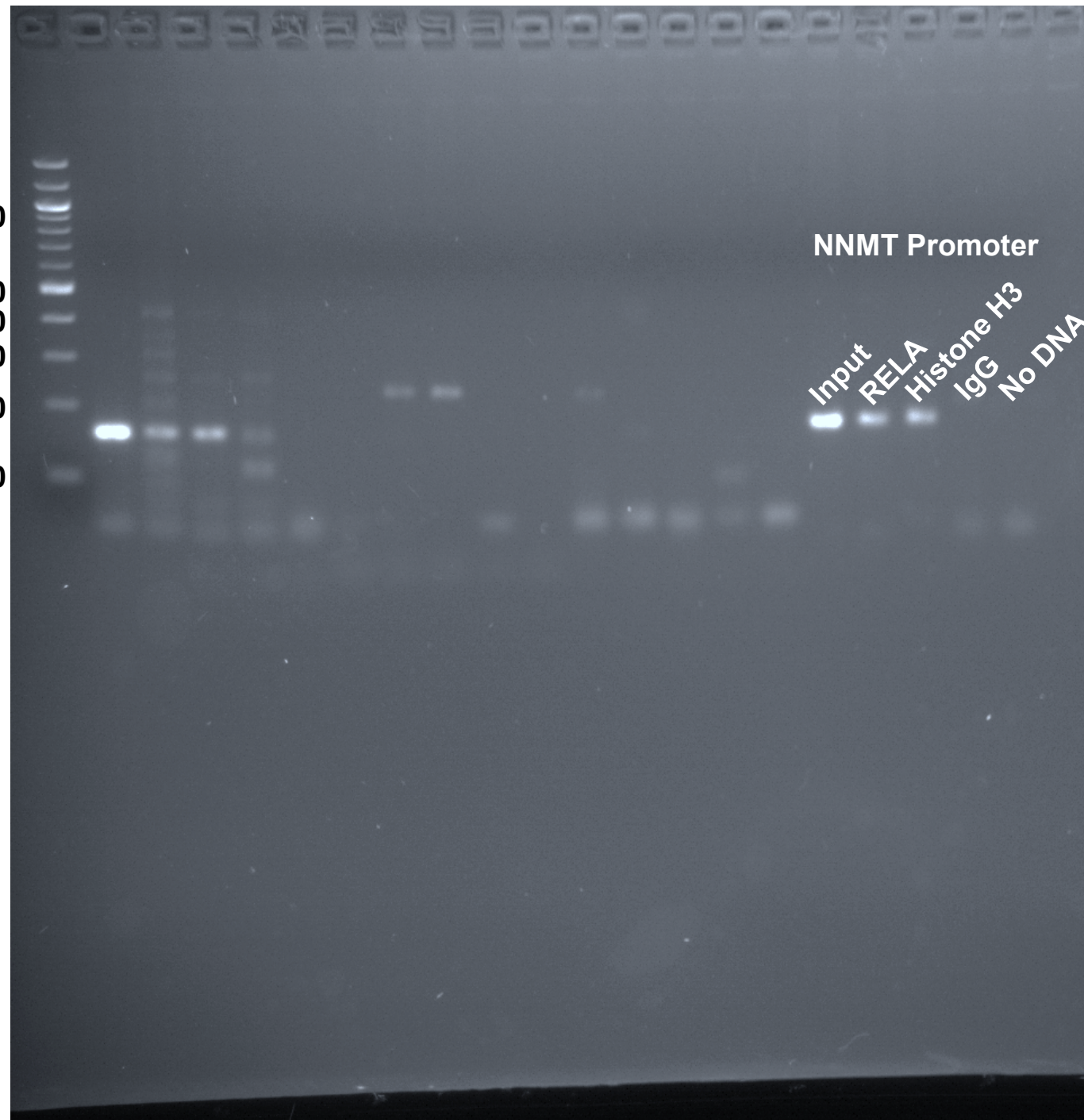

Supplement: Supplementary file 8 — Source Data Fig. 7 [file 44318_2023_9_MOESM8_ESM.zip › Figure 7/7E/Image-agarose gel.pdf]

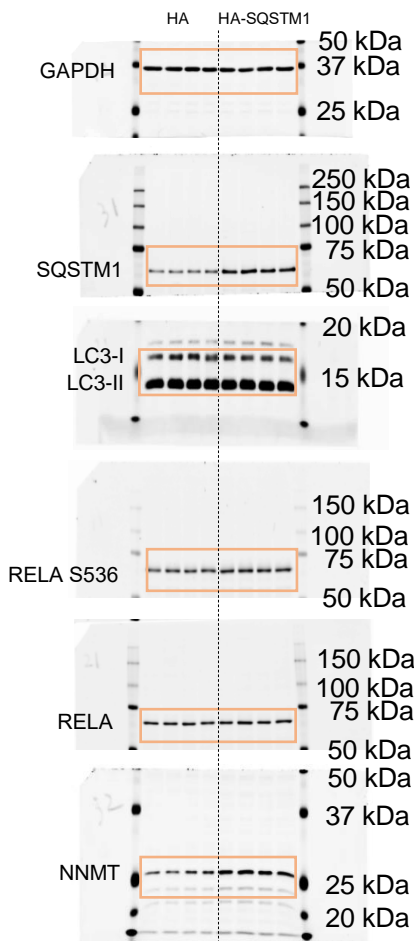

Supplement: Supplementary file 8 — Source Data Fig. 7 [file 44318_2023_9_MOESM8_ESM.zip › Figure 7/7K/Image-Blots.pdf]

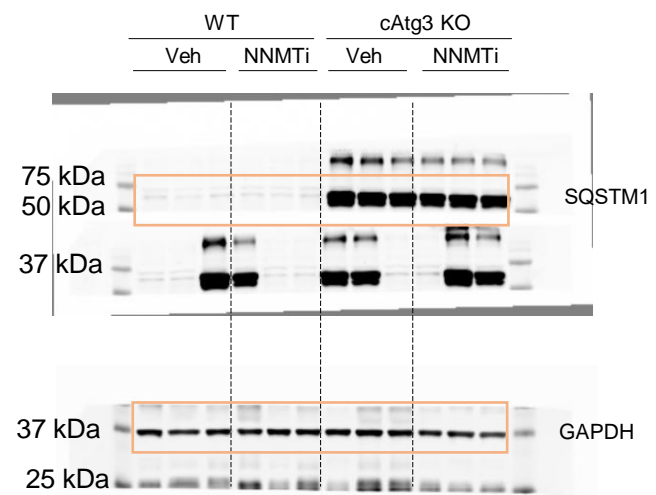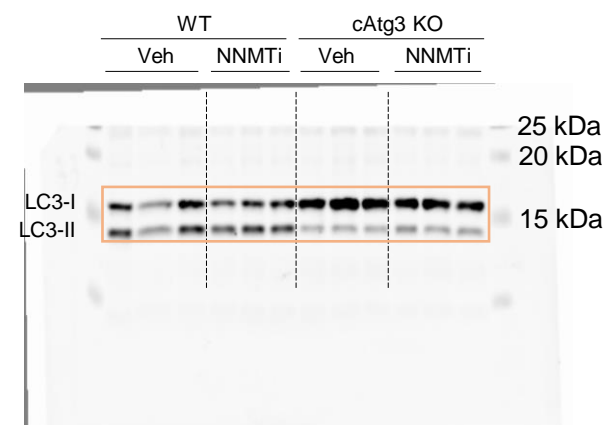

Supplement: Supplementary file 9 — Source Data Fig. 8 [file 44318_2023_9_MOESM9_ESM.zip › Figure 8/8F/Image-Blots.pdf]

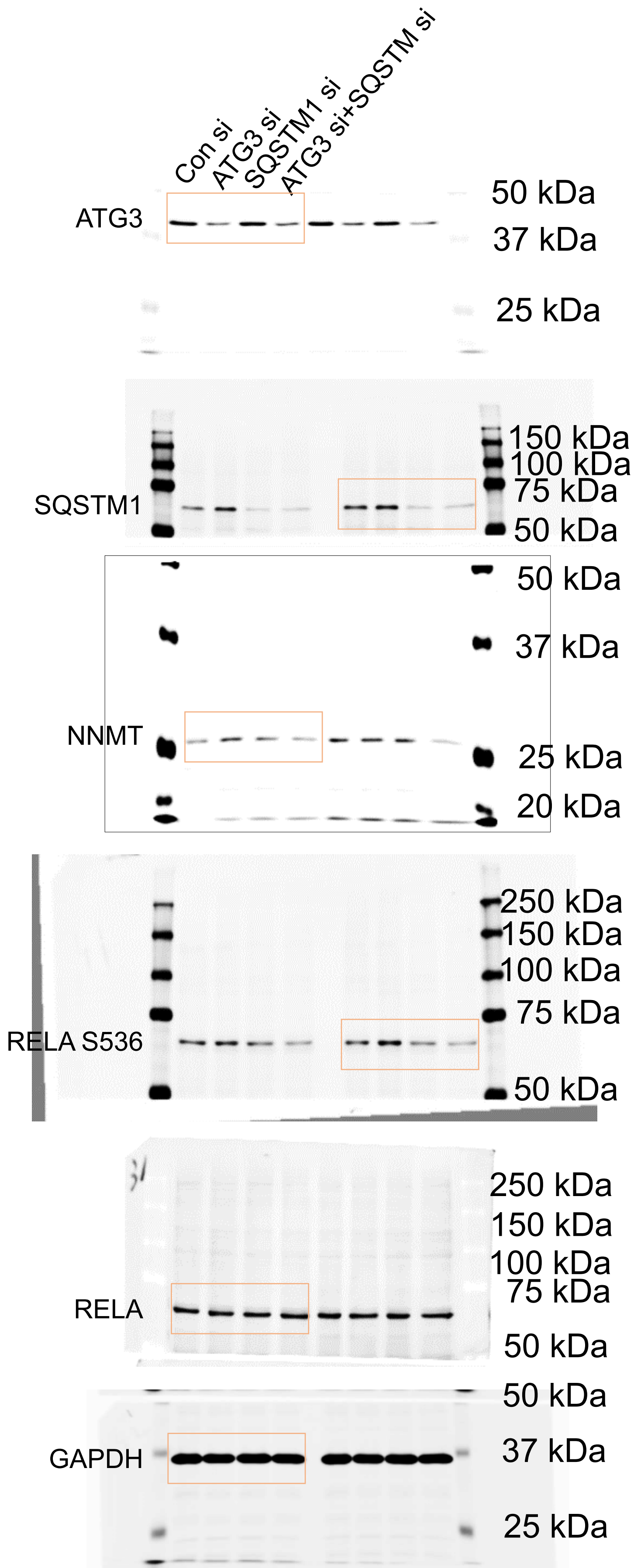

Supplement: Supplementary file 9 — Source Data Fig. 8 [file 44318_2023_9_MOESM9_ESM.zip › Figure 8/8A/Image-Blots.pdf]

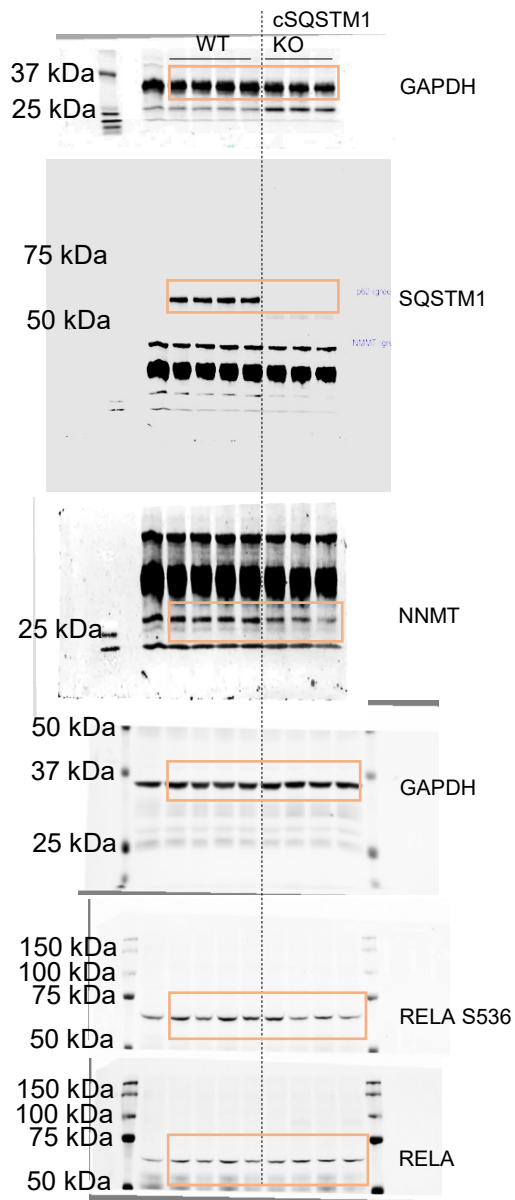

Supplement: Supplementary file 9 — Source Data Fig. 8 [file 44318_2023_9_MOESM9_ESM.zip › Figure 8/8D/8D Image-Blots.pdf]

S1A

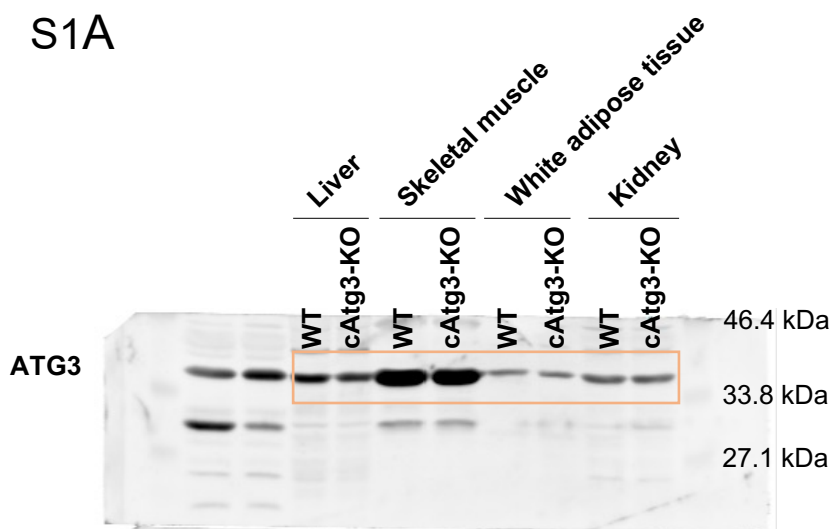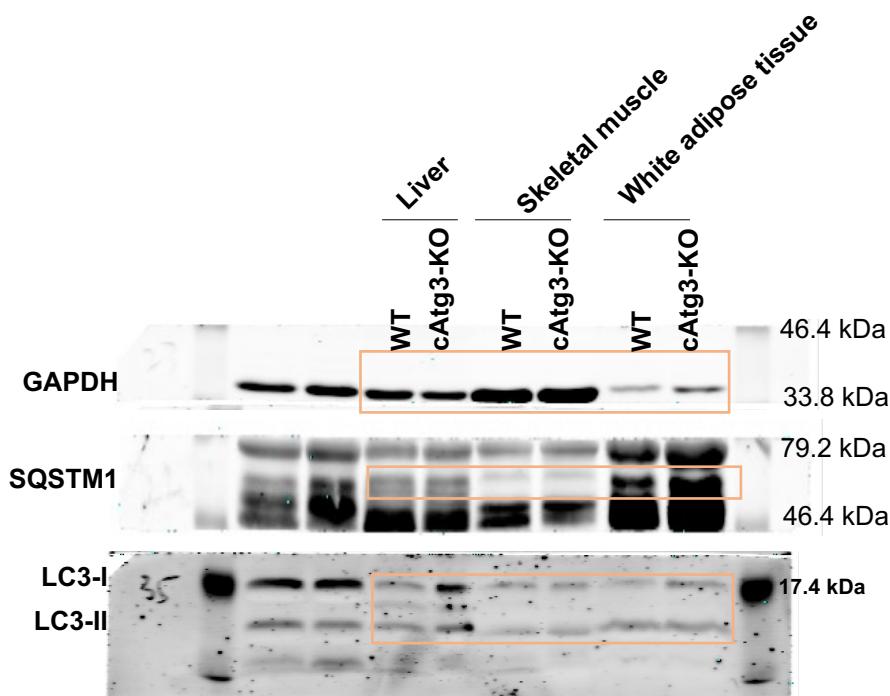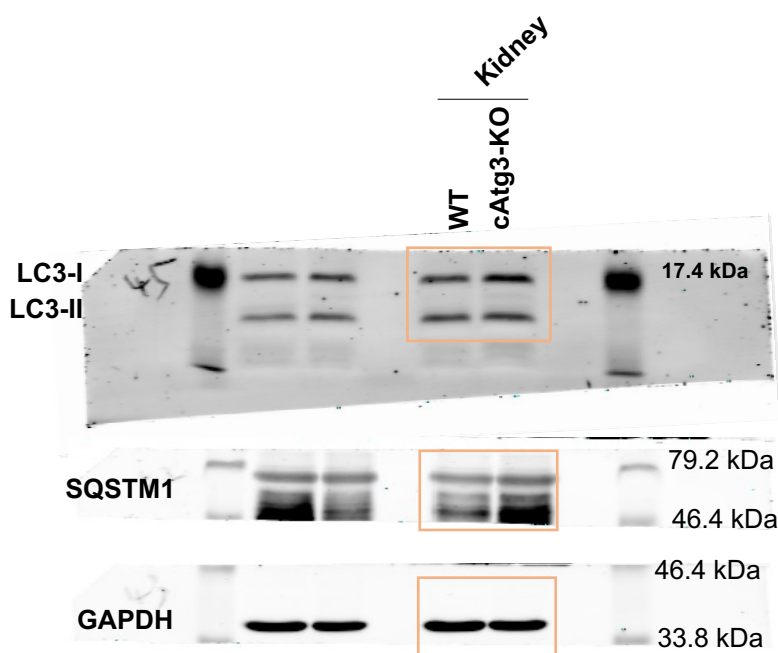

Supplement: Supplementary file 10 — EV and Appendix Figure Source Data [file 44318_2023_9_MOESM10_ESM.zip › Expanded source data/Appendix Figure S1/S1 A/S1A Image data, Blot..pdf]

S1B

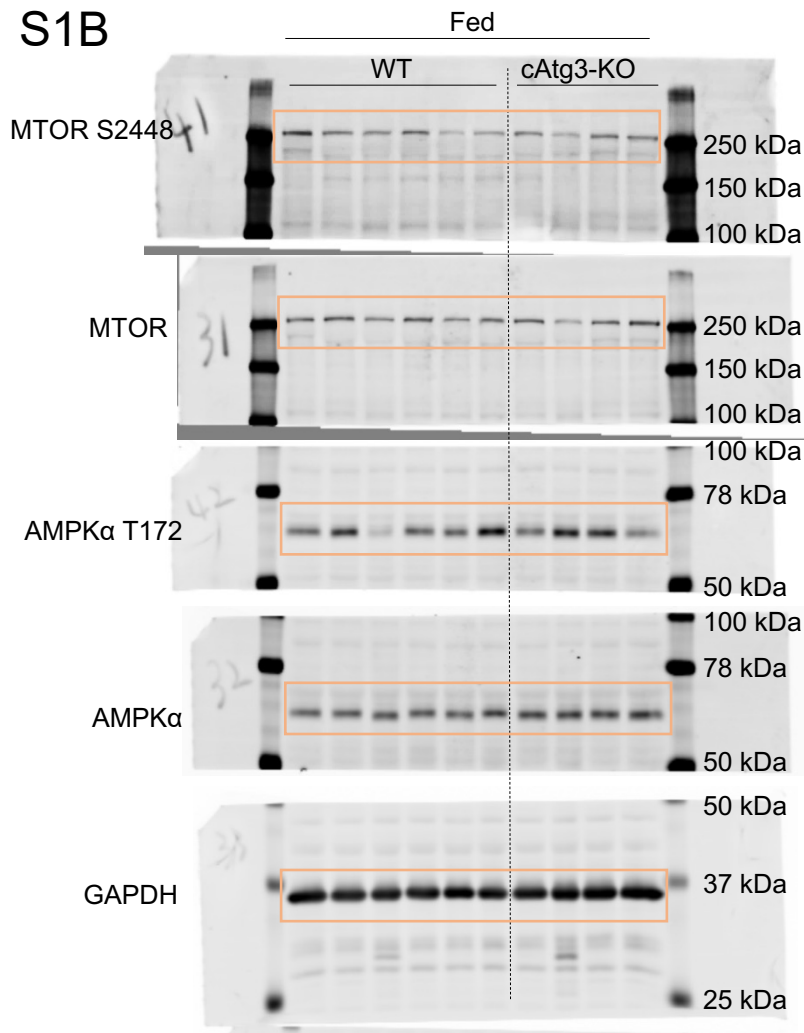

Supplement: Supplementary file 10 — EV and Appendix Figure Source Data [file 44318_2023_9_MOESM10_ESM.zip › Expanded source data/Appendix Figure S1/S1 B/S1B Image data, Blot.pdf]

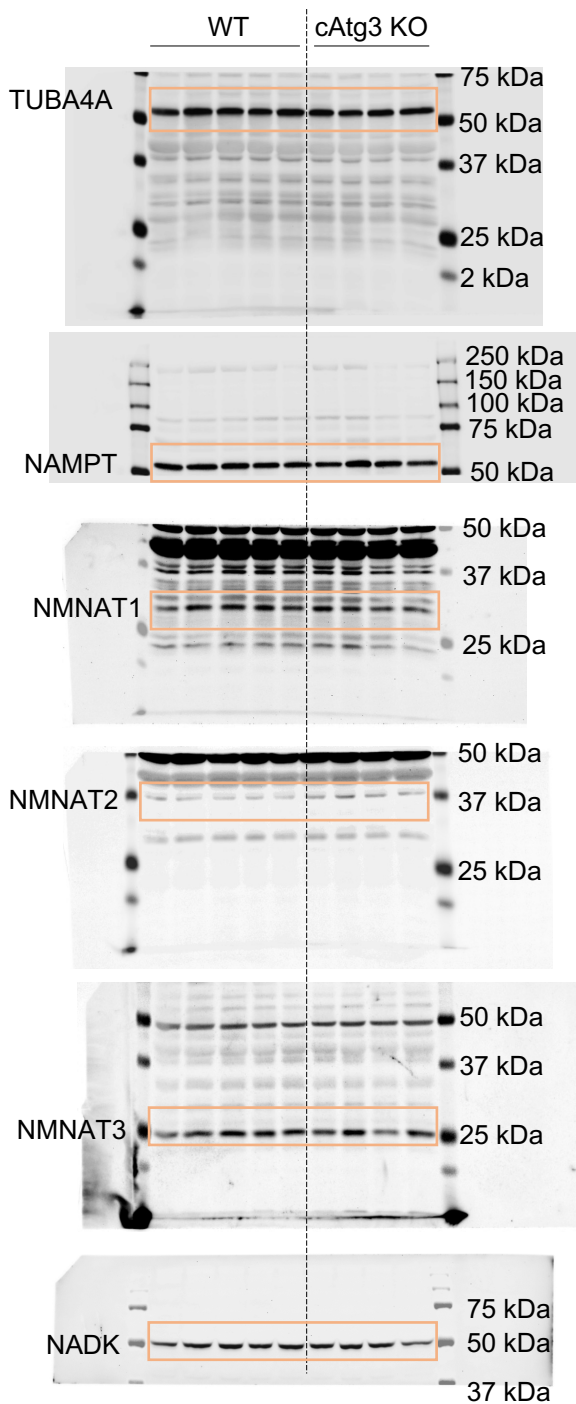

Supplement: Supplementary file 10 — EV and Appendix Figure Source Data [file 44318_2023_9_MOESM10_ESM.zip › Expanded source data/Appendix Figure S2/S2 Image data, Blot.pdf]

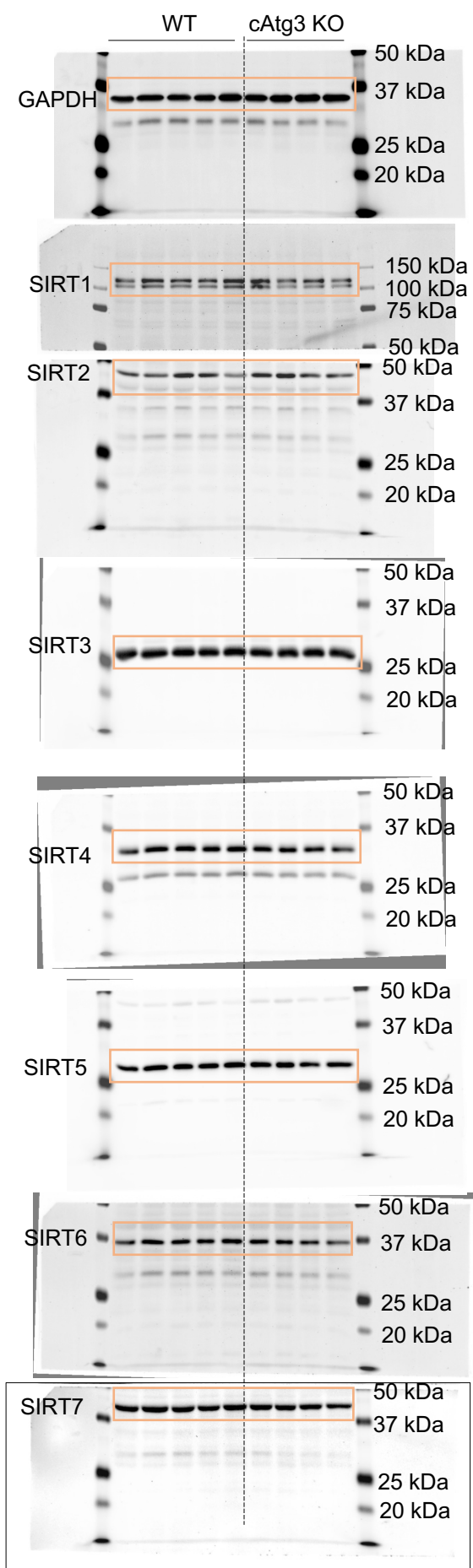

Supplement: Supplementary file 10 — EV and Appendix Figure Source Data [file 44318_2023_9_MOESM10_ESM.zip › Expanded source data/Appendix Figure S3/S3 A/S3A Image data, Blot.pdf]

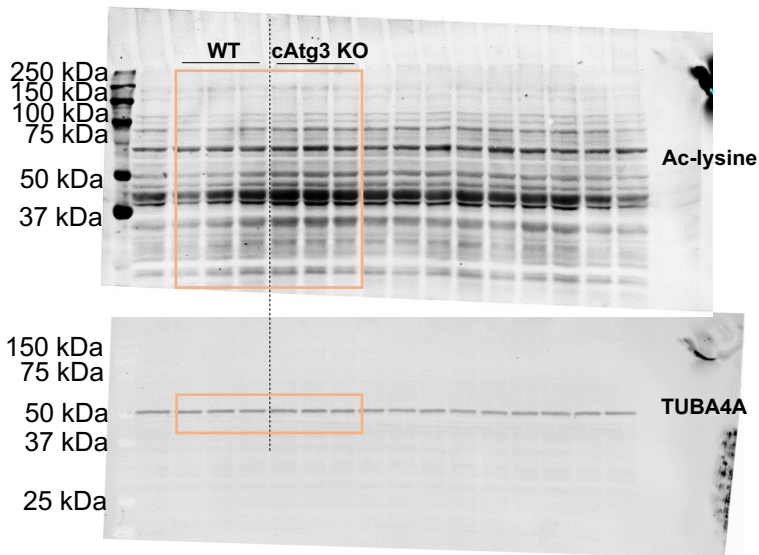

Supplement: Supplementary file 10 — EV and Appendix Figure Source Data [file 44318_2023_9_MOESM10_ESM.zip › Expanded source data/Appendix Figure S3/S3 C/S3 C Image data, Blot.pdf]

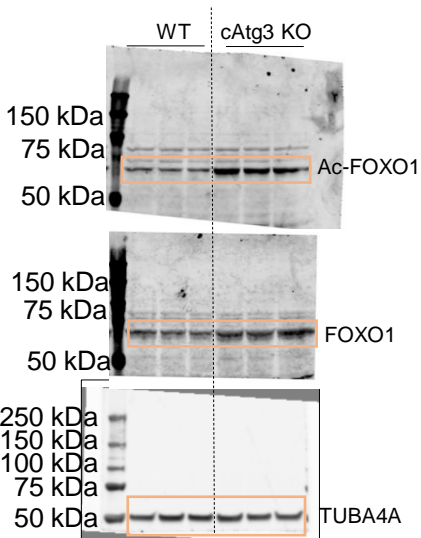

Supplement: Supplementary file 10 — EV and Appendix Figure Source Data [file 44318_2023_9_MOESM10_ESM.zip › Expanded source data/Appendix Figure S3/S3 D/S3 D Image data, Blot.pdf]

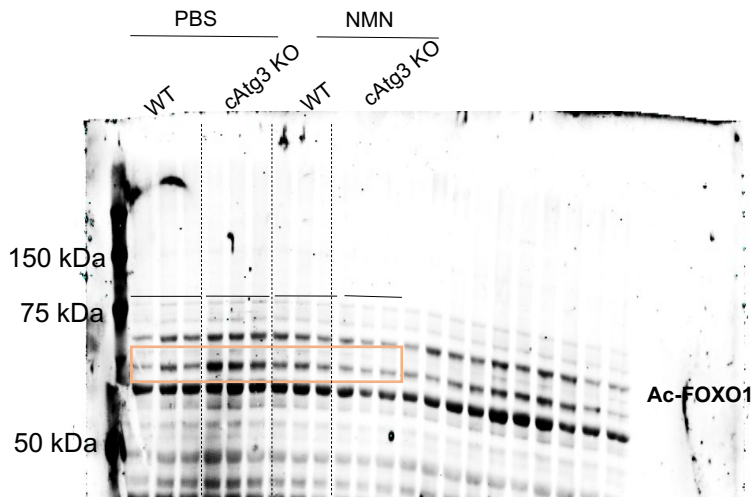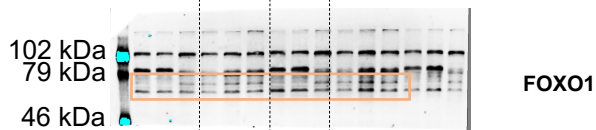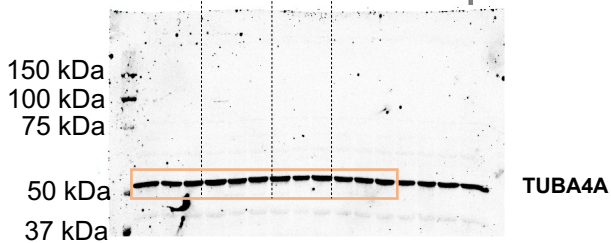

Supplement: Supplementary file 10 — EV and Appendix Figure Source Data [file 44318_2023_9_MOESM10_ESM.zip › Expanded source data/Appendix Figure S3/S3 E/S3 E Image data, Blot.pdf]

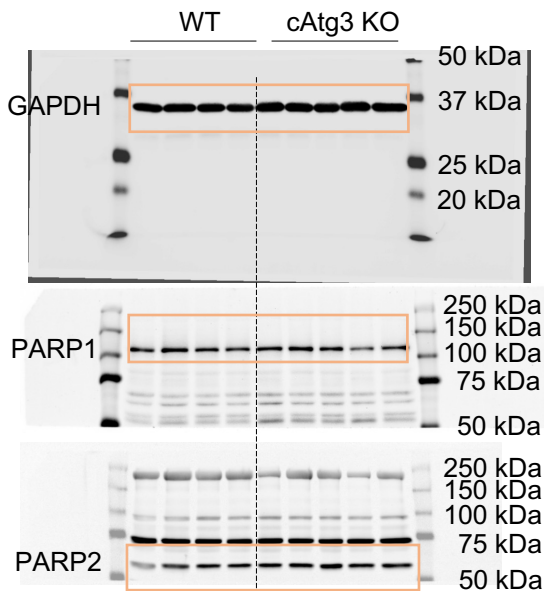

Supplement: Supplementary file 10 — EV and Appendix Figure Source Data [file 44318_2023_9_MOESM10_ESM.zip › Expanded source data/Appendix Figure S4/S4 A/S4 A Image data, Blot.pdf]

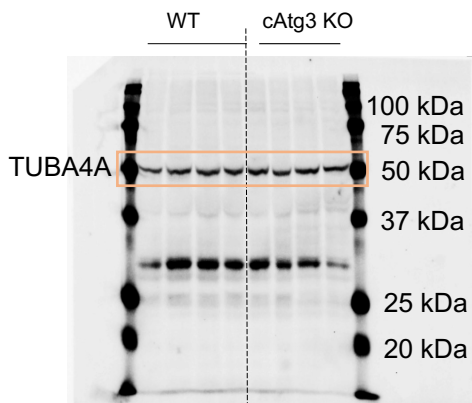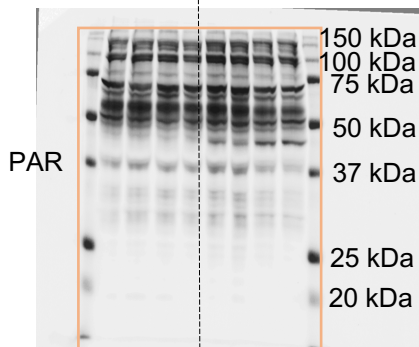

Supplement: Supplementary file 10 — EV and Appendix Figure Source Data [file 44318_2023_9_MOESM10_ESM.zip › Expanded source data/Appendix Figure S4/S4 C/S4 C Image, blot.pdf]

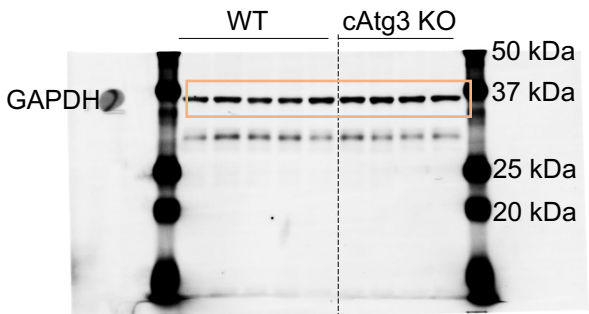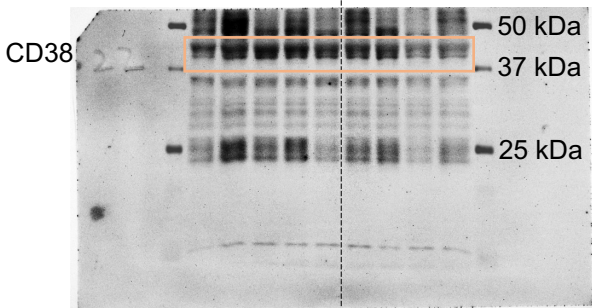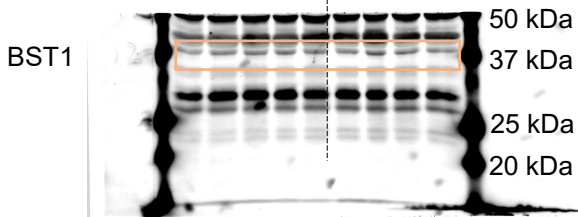

Supplement: Supplementary file 10 — EV and Appendix Figure Source Data [file 44318_2023_9_MOESM10_ESM.zip › Expanded source data/Appendix Figure S4/S4 D/S4 D Image data, Blot.pdf]

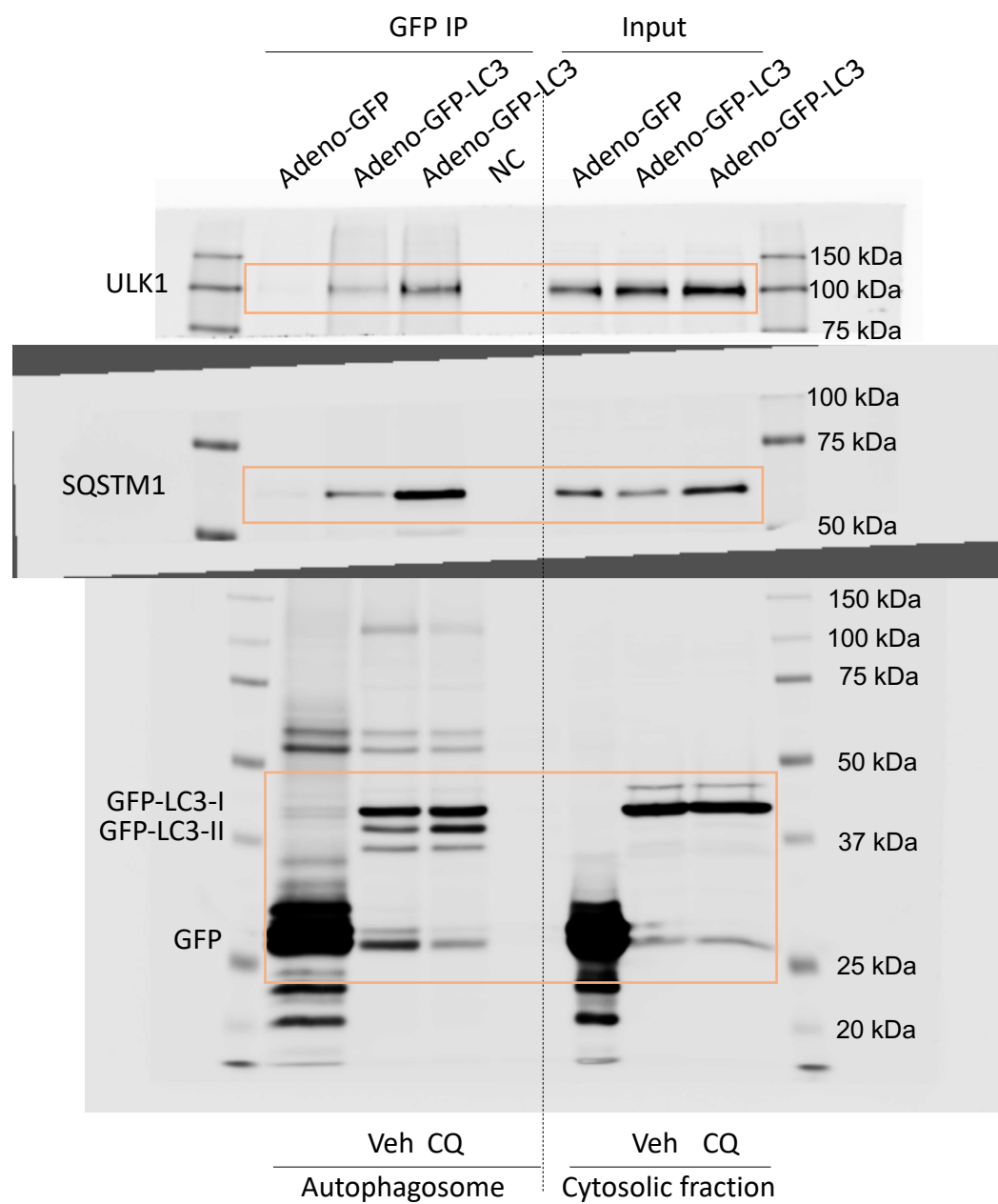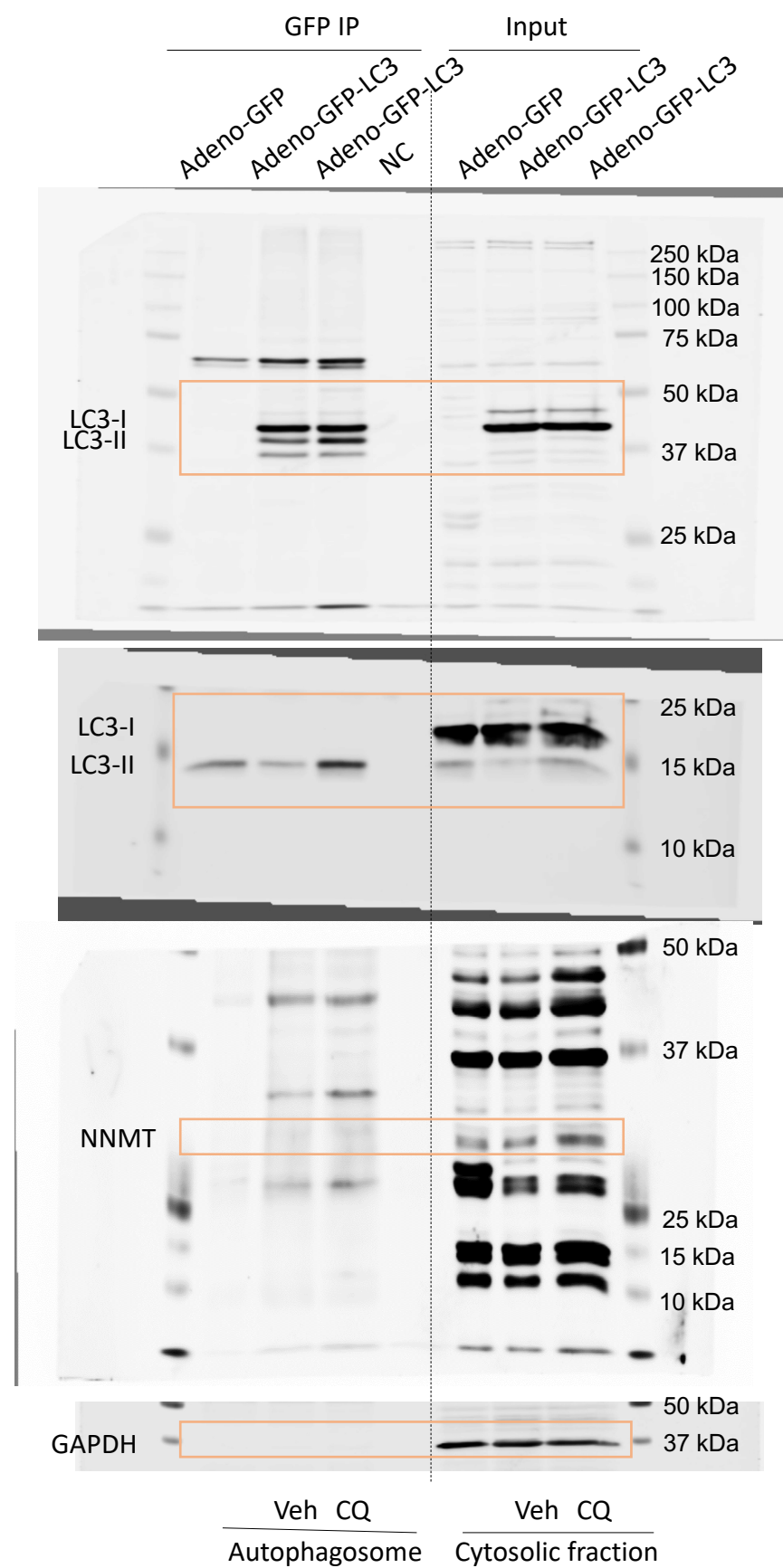

Supplement: Supplementary file 10 — EV and Appendix Figure Source Data [file 44318_2023_9_MOESM10_ESM.zip › Expanded source data/Appendix Figure S6/S6 A/S6 A Image data, Blot.pdf]

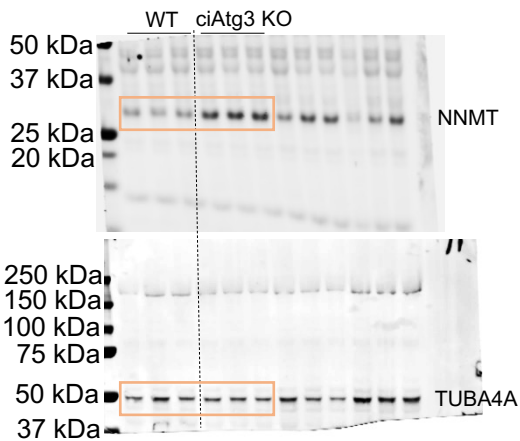

Supplement: Supplementary file 10 — EV and Appendix Figure Source Data [file 44318_2023_9_MOESM10_ESM.zip › Expanded source data/Expanded view figure 4/EV4 A/EV4 A Image data, Blot.pdf]

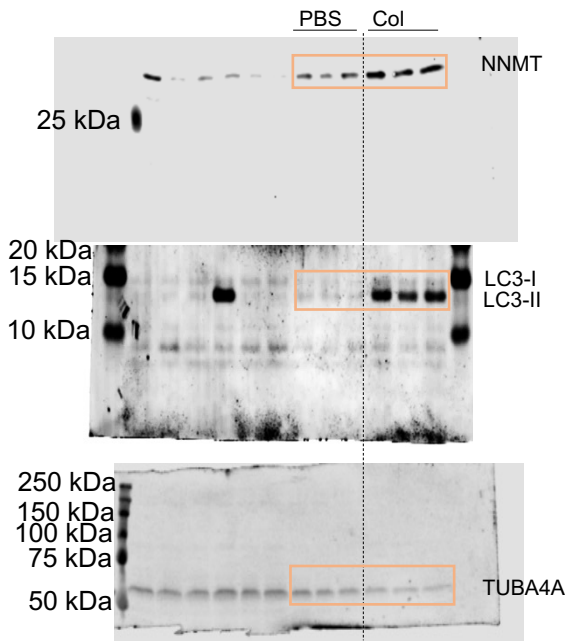

Supplement: Supplementary file 10 — EV and Appendix Figure Source Data [file 44318_2023_9_MOESM10_ESM.zip › Expanded source data/Expanded view figure 4/EV4 C/EV4 C Image data, Blot.pdf]

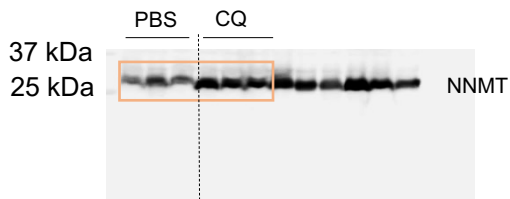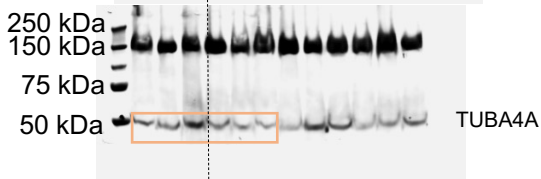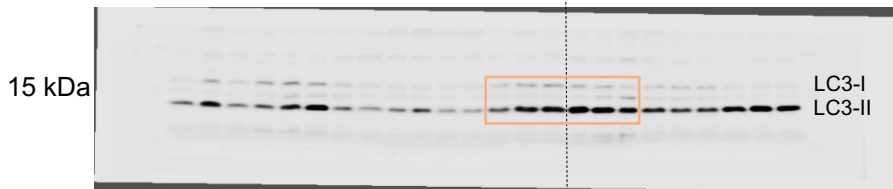

Supplement: Supplementary file 10 — EV and Appendix Figure Source Data [file 44318_2023_9_MOESM10_ESM.zip › Expanded source data/Expanded view figure 4/EV4 E/EV4 E Image data, Blot.pdf]

A

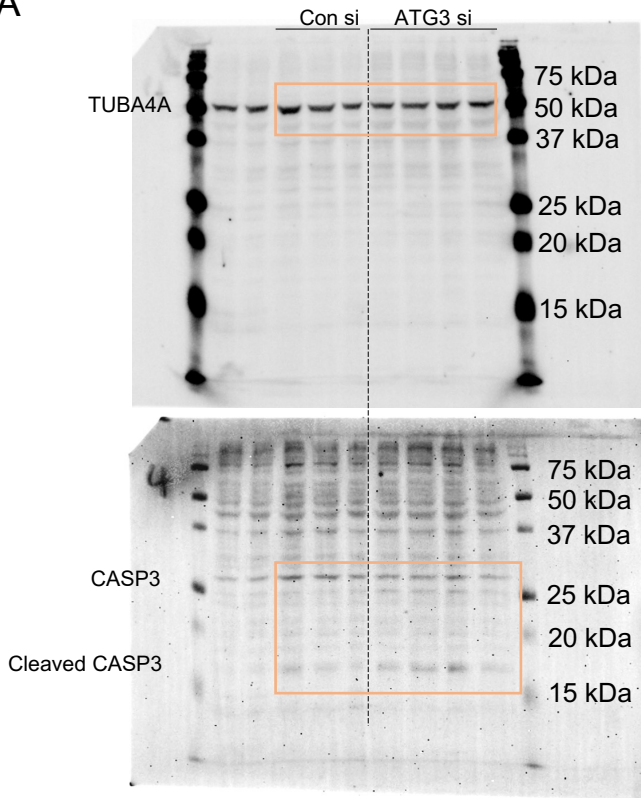

Supplement: Supplementary file 10 — EV and Appendix Figure Source Data [file 44318_2023_9_MOESM10_ESM.zip › Expanded source data/Expanded view figure 5/EV5 A/EV5 A Image data, Blot.pdf]

C

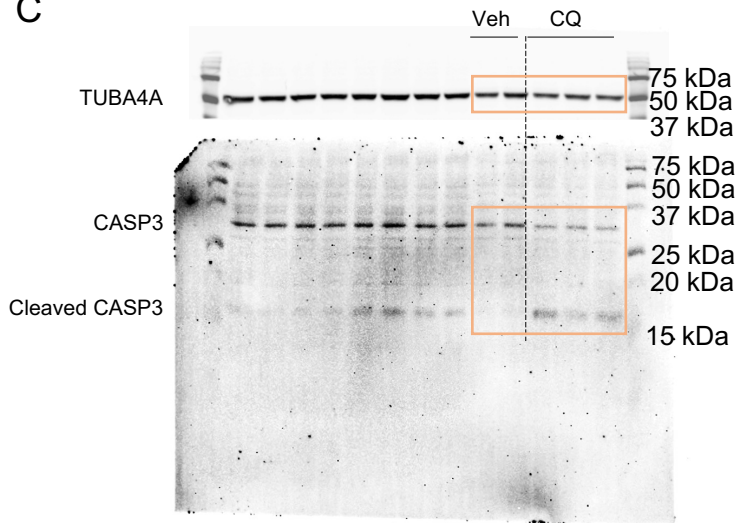

Supplement: Supplementary file 10 — EV and Appendix Figure Source Data [file 44318_2023_9_MOESM10_ESM.zip › Expanded source data/Expanded view figure 5/EV5 C/EV5 C Image data, Blot.pdf]
