# Supplementary material for: Control of NAD+ homeostasis by autophagic flux modulates mitochondrial and cardiac function
Source: EMBO J. 2024 Jan 11;43(3):3. doi: 10.1038/s44318-023-00009-w (PMC10897141; doi:10.1038/s44318-023-00009-w)
Supplement: Supplementary file 12 — Expanded View Figures [file 44318_2023_9_MOESM12_ESM.pdf]

Expanded View Figures

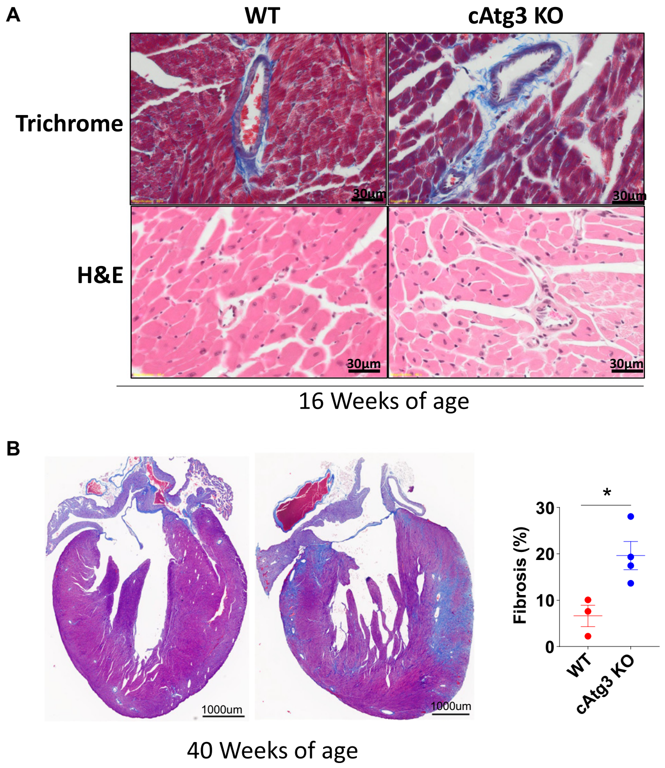

Figure EV1. CAtg3-KO develops fibrosis in the heart with aging, related to Fig. 1.

(**A**) Histology (Trichrome and Hematoxylin and Eosin (H&E)) of WT and cAtg3-KO mouse hearts at 16 weeks of age, $n = 6$ per group. Representative images are shown. (**B**) Histology (Trichrome and Hematoxylin and Eosin (H&E)) of WT and cAtg3-KO mouse hearts at 40 weeks of age, $n = 3$–4 per group. Data are mean ± SEM. An unpaired t test was used to determine statistical significance between two groups. $^{*}P < 0.05$. Source data are available online for this figure.

 © The Author(s)

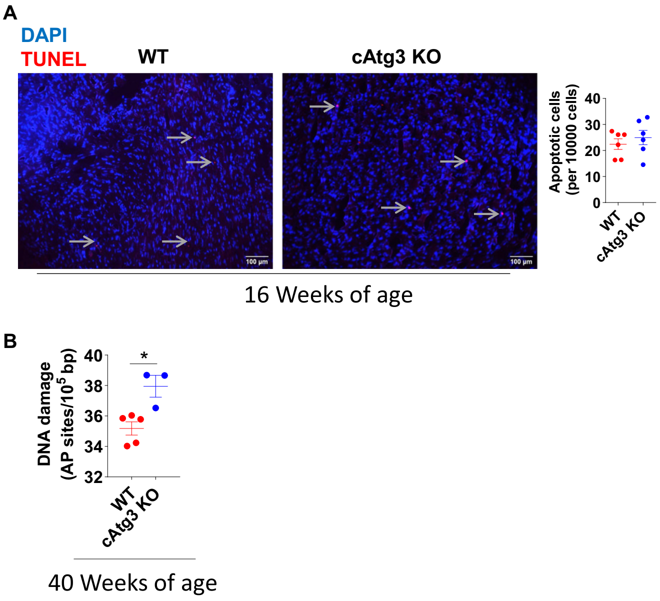

Figure EV2. Apoptosis and DNA damage in cAtg3-KO with aging, related to Fig. 1.

(**A**) TUNEL staining in WT and cAtg3-KO mouse hearts. Sections were obtained from mice at 16 weeks of age, $n = 6$ per group. Arrows represent TUNEL-positive nuclei. Data are mean ± SEM. (**B**) DNA damage in WT and cAtg3-KO mouse hearts at 40 weeks of age. $n = 3$–5 per group. Data are mean ± SEM. An unpaired t test was used to determine statistical significance between two groups. *$P < 0.05$. Source data are available online for this figure.

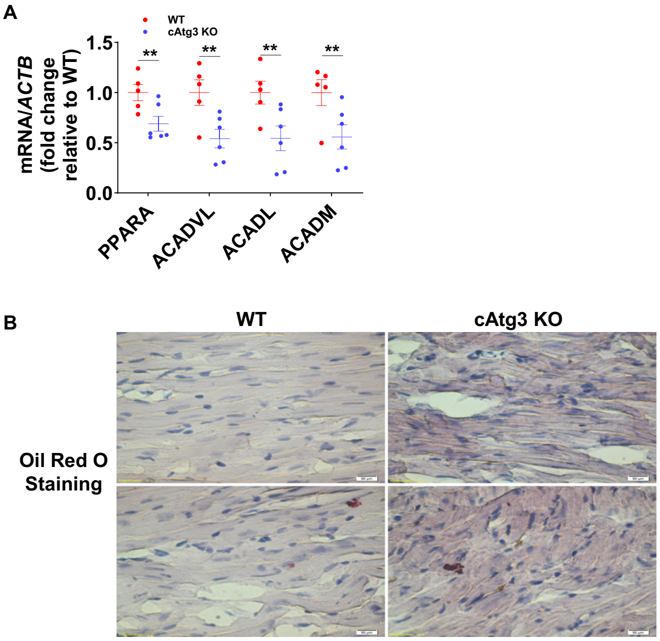

Figure EV3. Decreased expression of fatty acid oxidation genes and increased lipid accumulation in cAtg3-KO hearts, related to Fig. 2.

(**A**) mRNA levels of fatty acid oxidation related genes in 16-week-old WT and cAtg3-KO mouse hearts. $n = 4$–6. Data are mean ± SEM. Unpaired t tests were used to determine statistical significance between two groups. $^{**}P < 0.01$. (**B**) Representative oil red O staining of tissue sections from WT and cAtg3-KO mouse hearts at 16 weeks of age, $n = 2$ per group. Representative section images are shown. Source data are available online for this figure.

 © The Author(s)

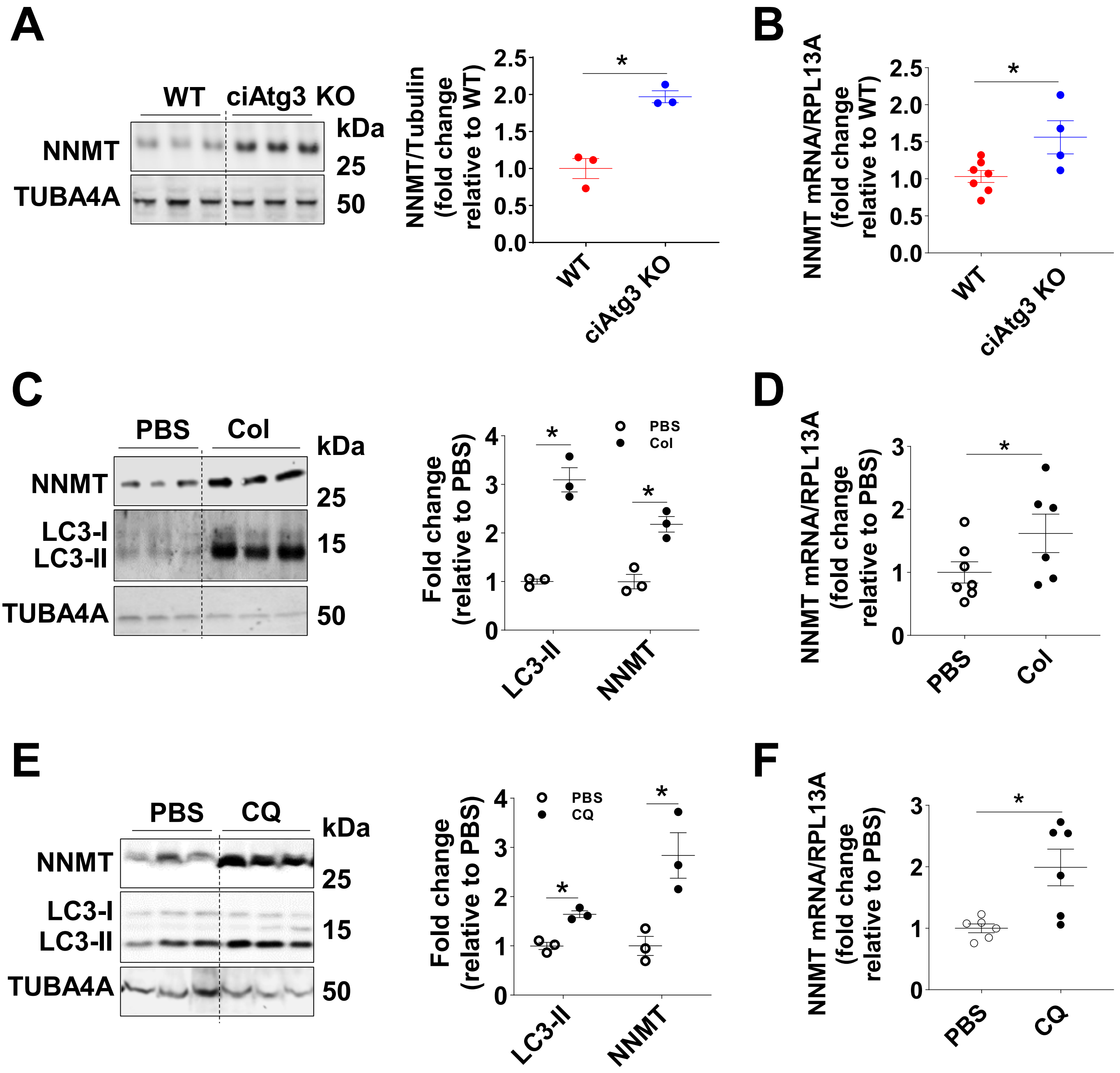

Figure EV4. CAtg3-KO upregulates NNMT expression in hearts, related to Fig. 5.

(**A**) NNMT protein levels in WT and ciAtg3 KO mice 1 week after last tamoxifen injection. $n = 3$ per group. Data are mean ± SEM. An unpaired t test was used to determine statistical significance between two groups. *$P < 0.05$. (**B**) *NNMT* mRNA levels in WT and ciAtg3 KO mice 1 week after last tamoxifen injection. $n = 4$–7 per group. Data are mean ± SEM. An unpaired t test was used to determine statistical significance between two groups. *$P < 0.05$. (**C**) NNMT and LC3 (MAP1LC3A) protein levels in hearts of WT mice following treatment with PBS, or Colchicine (Col), $n = 3$ per group. Data are mean ± SEM. An unpaired t test was used to determine statistical significance between two groups. *$P < 0.05$. (**D**) *NNMT* mRNA levels in hearts of WT mice following treatment with PBS, or Colchicine (Col). $n = 6$–7 per group. Data are mean ± SEM. An unpaired t test was used to determine statistical significance between two groups. *$P < 0.05$. (**E**) NNMT and LC3 protein levels in hearts of WT mice following treatment with PBS, or Chloroquine (CQ). $n = 3$ per group. Data are mean ± SEM. An unpaired t test was used to determine statistical significance between two groups. *$P < 0.05$. (**F**) *NNMT* mRNA levels in hearts of WT mice following treatment with PBS, or CQ. $n = 6$ per group. Data are mean ± SEM. An unpaired t test was used to determine statistical significance between two groups. *$P < 0.05$. Source data are available online for this figure.

© The Author(s)

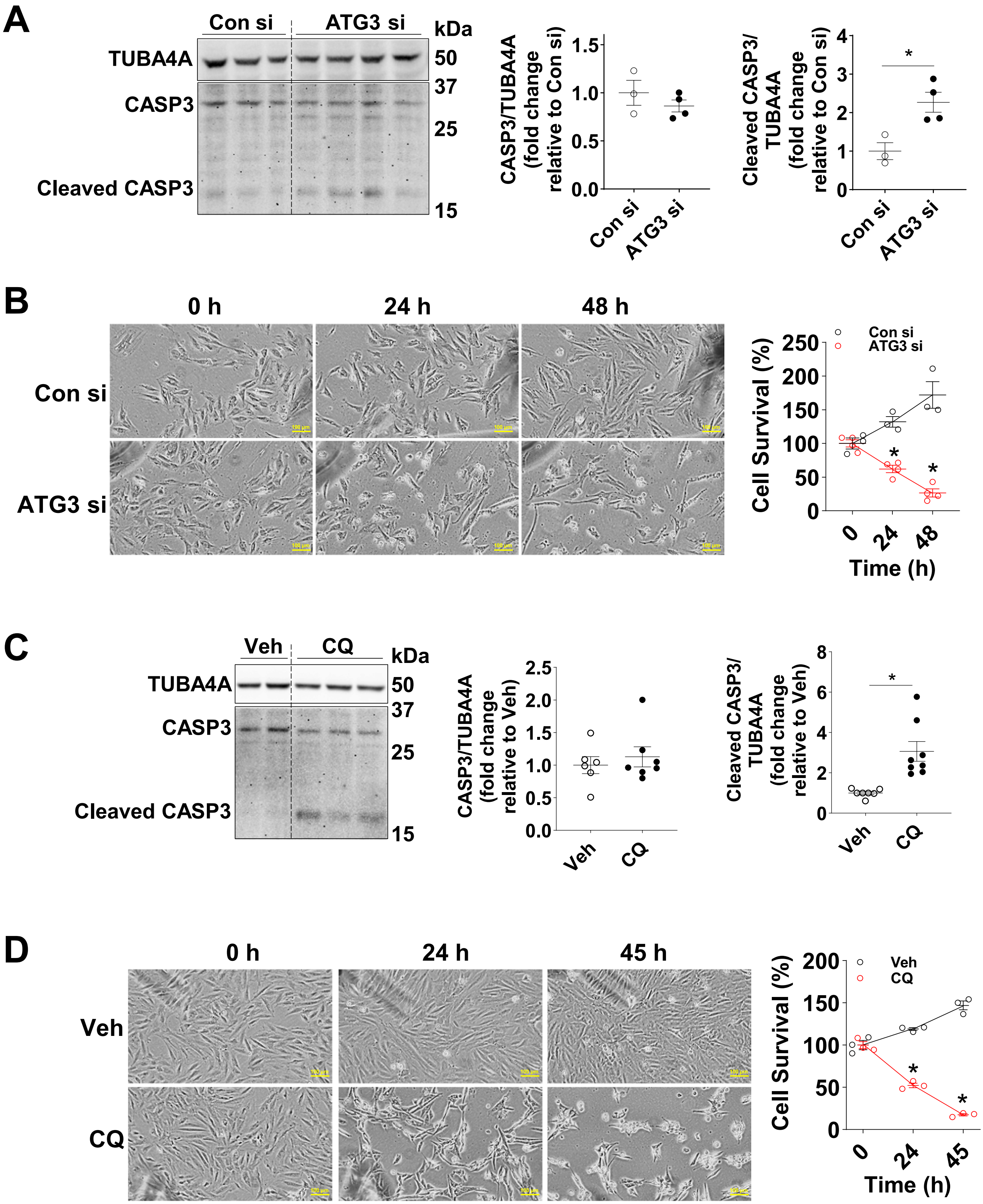
A
Con si
ATG3 si
kDa
TUBA4A
50
37
CASP3
25
Cleaved CASP3
15
CASP3/TUBA4A (fold change relative to Con si)
1.5
1.0
0.5
0.0
Con si
ATG3 si
Cleaved CASP3/ TUBA4A (fold change relative to Con si)
4
3
2
1
0
*
Con si
ATG3 si
B
0 h
24 h
48 h
Con si
ATG3 si
Cell Survival (%)
250
200
150
100
50
0
Con si
ATG3 si
*
*
0
24
48
Time (h)
C
Veh
CQ
kDa
TUBA4A
50
37
CASP3
25
Cleaved CASP3
15
CASP3/TUBA4A (fold change relative to Veh)
2.5
2.0
1.5
1.0
0.5
0.0
Veh
CQ
Cleaved CASP3/ TUBA4A (fold change relative to Veh)
8
6
4
2
0
*
Veh
CQ
D
0 h
24 h
45 h
Veh
CQ
Cell Survival (%)
200
150
100
50
0
Veh
CQ
*
*
0
24
45
Time (h)

 © The Author(s)

Figure EV5. Cell death under conditions of defective autophagy, related to Fig. 6.

(**A**) Protein levels of CASP3 (Caspase3), Cleaved CASP3, and TUBA4A (alpha-tubulin) in H9c2 cells transfected with *ATG3* siRNA. *ATG3* siRNA was added to cells for ~48 h prior to harvesting cells for western blot. $n = 3$–4 per group. Data are mean ± SEM. Unpaired *t* tests were used to determine statistical significance between two groups. *$P < 0.05$. (**B**) Cell death in H9c2 cells cultured in nutrient-replete medium. H9c2 cells were transfected with *ATG3* siRNA for ~48 h. Then, the cells were moved to the growth medium (high-glucose DMEM supplemented with 10% FBS), and the images in the same location in each well were captured at the end of siRNA transfection (T0) and then at 24 h and 48 h thereafter. $n = 3$–4 images at each time point per group. Data are mean ± SEM. Unpaired *t* tests were used to determine statistical significance between two groups at corresponding time points. *$P < 0.05$ vs. Con si-treated H9c2 cells at the corresponding time points. (**C**) Protein levels of CASP3, Cleaved CASP3, and TUBA4A in H9c2 cells treated with 20 μM chloroquine (CQ) for 16 h. $n = 6$–8 per group. Data are mean ± SEM. Unpaired *t* tests were used to determine statistical significance between two groups. *$P < 0.05$. (**D**) Cell death in H9c2 cells cultured in nutrient-replete medium. H9c2 cells were treated with 20 μM CQ. There was no evidence of cell death at 16 h. However, cell viability is reduced at 24 h. Images of H9c2 cells in the same location in each well were captured at time points indicated, followed by cell counting. $n = 3$ images at each time point per group. Data are mean ± SEM. Unpaired *t* tests were used to determine statistical significance between two groups at corresponding time points. *$P < 0.05$ vs. Veh-treated H9c2 cells at the corresponding time points. Source data are available online for this figure.

© The Author(s)
